# Optimized ensemble machine learning model for cyberattack classification in industrial IoT

**DOI:** 10.3389/frai.2025.1685376

**Published:** 2026-01-12

**Authors:** Batool Alabdullah, Suresh Sankaranarayanan

**Affiliations:** College of Computer Sciences and Information Technology, Department of Computer Science, King Faisal University, Al-Ahsa, Saudi Arabia

**Keywords:** cyberattack, ensemble learning, industrial control systems, industrial internet of things, internet of things, machine learning, malicious behavior, oil and gas

## Abstract

**Introduction:**

The increasing cyber threats targeting industrial control systems (ICS) and the Internet of Things (IoT) pose significant risks, especially in critical infrastructures like the oil and gas sector. Existing machine learning (ML) approaches for cyberattack detection often rely on binary classification and lack computational efficiency.

**Methods:**

This study proposes two optimized stacked ensemble models to enhance attack detection accuracy while reducing computational overhead. The main contribution lies in the strategic selection and integration of diverse base models, such as Logistic Regression, Extra Tree Classifier, XGBoost, and LGBM, with RFC as the final estimator. These models are chosen to address unique characteristics of security datasets, such as class imbalance, noise, and complex attack patterns. This combination aims to leverage different decision boundaries and learning mechanisms.

**Results:**

Evaluations show that the Stacked Ensemble_2 model achieves 97% accuracy with a training and testing computation time of 54 minutes. Stacked Ensemble_2, which excelled over the traditional Stacked Ensemble_1, was also evaluated on the CICIDS 2017 dataset, achieving an impressive 100% accuracy with an AUROC of 99%.

**Discussion:**

The results indicate that the proposed Stacked Ensemble_2 model provides a scalable, real-time detection mechanism for securing ICS and IoT environments. By proving its effectiveness on unseen data, this model demonstrates a significant advancement over traditional methods, offering enhanced accuracy and efficiency in detecting sophisticated cyber threats in critical infrastructure sectors.

## 1 Introduction

The rapid evolution of IoT and ICS technologies has dramatically reshaped business and government functions. By facilitating automation, real-time monitoring, and data-driven decision-making, these technologies significantly enhance critical infrastructure. However, the increased connectivity they bring also poses substantial cybersecurity challenges, highlighting the necessity for organizations worldwide to prioritize the protection of industrial operations and sensitive data. According to the IoT Analytics “State of IoT—Spring 2023” report, the number of IoT devices surged by 18% in 2022, totaling about 14.4 billion active connections. These devices, including sensors, actuators, and communication modules, enable seamless data exchange across various industries, driving crucial applications in fields such as healthcare, manufacturing, and energy (Sinha, 2024; Global Market Insights, Inc., 2019).

The advent of the Fourth and Fifth Industrial Revolutions has resulted in greater connectivity between ICS and IoT systems, enabling features such as remote monitoring, automation, and cloud-based control. Despite these advancements, traditional cybersecurity tools—like antivirus programs, firewalls, and Intrusion Detection Systems—are often insufficient for detecting advanced and sophisticated cyber threats targeting these systems. In the oil and gas sector, IoT and ICS technologies are employed for tasks such as pipeline vandalism detection, digital twin development, reservoir evaluation, and methane gas monitoring. However, cybersecurity efforts in this industry have not received enough emphasis. Considering the sector’s critical importance to the global economy, cyberattacks on oil and gas infrastructure can lead to serious repercussions, including operational disruptions, data manipulation, and significant financial damage (Ghosh et al., 2022; Lukman, 2018; Ochulor, 2024; Knebel et al., 2023; Ali, 2024).

The economic impact of cyberattacks on critical infrastructure is substantial. Studies utilizing large language models (LLMs) for cyberattack cost estimation highlight the significant financial burdens resulting from security breaches (Razavi and Jamil, 2024), while research on big data analytics in banking cybersecurity shows how attacks can lead to long-term financial and reputational damage (Razavi et al., 2023). These insights underscore the urgent need for robust cybersecurity frameworks capable of proactively detecting and mitigating threats within industrial environments.

Currently, most machine learning (ML) methods for ICS and IoT security are limited to binary classification, which differentiates between normal and malicious traffic. However, real-world cyber threats are often varied and complex, necessitating multi-class classification models that can accurately identify and categorize different types of attacks. Overcoming this challenge involves employing advanced techniques such as feature engineering, hyperparameter optimization, and ensemble learning to enhance detection accuracy and enable real-time threat response (Alsolami et al., 2024).

Despite the crucial role of the oil and gas industry in the global economy, cybersecurity in this sector has not received sufficient attention. While some research investigates protective strategies for ICS and IoT systems, few studies explore comprehensive, machine learning-based threat detection specifically tailored for this domain. Given the potentially devastating financial and operational consequences of cyberattacks on critical infrastructure, developing advanced, multi-class classification models and strengthening cybersecurity frameworks are essential for safeguarding these vital industries.

This research aims to enhance cybersecurity in the oil and gas industry by developing an optimized stacked ensemble-based machine learning model capable of detecting and classifying cyber threats into 15 attack categories, including:

- *DDoS attacks* (UDP, ICMP, HTTP, TCP)
- *Web-based attacks* (SQL Injection, XSS, Uploading)
- *Credential and access exploits* (Password Attacks, Backdoor, MITM)
- *Network and system vulnerabilities* (Port Scanning, Fingerprinting, Vulnerability Scanners)
- *Advanced threats* (Ransomware)

To improve detection accuracy, this study applies:

- *Feature engineering*: Encoding and selecting relevant attributes from the Edge-IIoTset dataset, such as IP addresses, frame timestamps, and URLs.
- *Feature selection*: Using variance thresholding to retain only the most impactful features.
- *Hyperparameter tuning*: Optimizing model parameters (e.g., kernel, gamma, C, Var smoothing, and weights) through Grid Search.
- *Ensemble learning optimization*: Developing a stacked ensemble model and comparing it with Bagging, Boosting, Extra Trees, and Random Forest Classifiers to achieve optimal performance.

The main contribution of our approach lies in the strategic selection and integration of diverse base models, including LightGBM, Extra Trees, and Logistic Regression, which are chosen to address the unique characteristics of security datasets, such as class imbalance, noise, and complex attack patterns. This curated combination aims to leverage different decision boundaries and learning mechanisms, thereby enhancing the ensemble’s ability to detect sophisticated threats effectively. Furthermore, our work demonstrates significant empirical improvements in attack detection accuracy, robustness, and interpretability—particularly important for practical cybersecurity deployment.

The inclusion of Logistic Regression, for instance, adds an interpretable component that provides insights into decision-making processes, aiding cybersecurity analysts in understanding attack behaviors. In summary, rather than presenting a generic stacking approach, our main focus is on the domain-informed design and extensive evaluation of these ensemble architectures in real-world ICS and IoT security contexts, offering insights and methodologies that can be adopted for similar cybersecurity challenges.

This study prioritizes detection over prevention, as cyber threats in ICS and IoT environments are constantly evolving. By enhancing detection capabilities, our approach minimizes the impact of cyberattacks, ensuring greater security for industrial operations and critical infrastructure. The model, optimized for high accuracy and efficient computation time, was tested on a new, unseen dataset. It achieved excellent performance, demonstrating the model’s strong ability to generalize effectively to previously unseen data.

The rest of the article is organized as follows. Sections 2 and 3 provide the background and a literature review relevant to this study. Section 4 details the implementation methodology, including dataset information, feature engineering, and feature selection techniques. Section 5 outlines the proposed approach, detailing the model architecture and optimized hyperparameter settings for various machine learning algorithms. Section 6 presents experimental results and analysis, comparing Stacked Ensemble_1 and Stacked Ensemble_2 with individual models such as Logistic Regression, dt, bagging, boosting, extra tree classifier, and RFC. Section 7 discusses the generalization of models on new unseen data, demonstrating the effectiveness of model performance in terms of the two stacked ensemble models. Section 8 benchmarks the proposed model against baseline approaches and existing research. Finally, Section 9 concludes with key findings and future research directions.

## 2 Background

The primary methods for exploring and extracting oil and gas resources in any country are onshore and offshore drilling. Onshore drilling involves deploying specialized equipment, platforms, and infrastructure in land-based environments to access subsurface resources. In contrast, offshore drilling occurs at considerable distances from the coast, with rigs operating in water depths ranging from about 10 feet to over 10,000 feet, introducing additional complexities to the drilling process (Mmrbh, 2022; Mohammed, 2024).

The adoption of Industrial Internet of Things (IIoT) technology connects intelligent industrial devices with control and management platforms to boost operational efficiency and productivity. However, this increased connectivity also exposes Industrial Control System (ICS) communication protocols to cyber threats, including data theft and malware infiltration.

**Figure 1:**
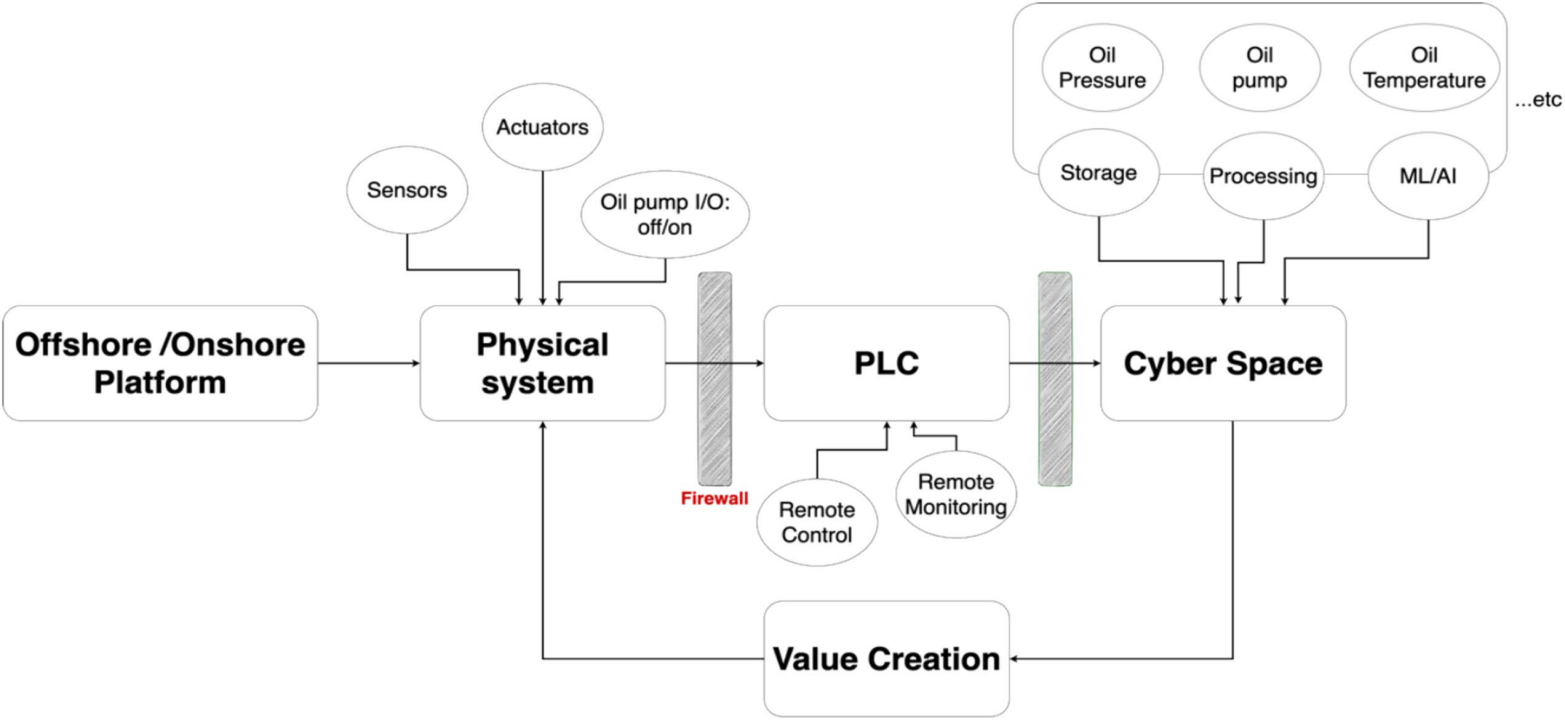
Example of typical components of ICPS in the oil and gas sector.

Industrial cyber-physical systems (ICPS) typically consist of three main control components: (1) programmable logic controllers (PLCs), (2) supervisory control and data acquisition (SCADA) systems, and (3) distributed control systems (DCS). The communication networks linking these components play a crucial role by connecting devices and equipment through various protocols, enabling efficient, system-wide communication. Nevertheless, this high level of interconnectedness makes ICPS attractive targets for cyberattacks aimed at disrupting critical operations. Figure 1 depicts a typical ICPS architecture in the oil and gas industry (Mohammed, 2024; Galloway and Hancke, 2012; Stouffer et al., 2015; Kayan et al., 2022).

Notably, some offshore platforms are now designed as unmanned facilities, requiring 100% remote monitoring and control through digital networks. This reliance on digital communication has introduced new security challenges for these remote and isolated offshore operations (Mohammed, 2024; Kristiansen and Mæland, 2020).

One common method attackers use to infiltrate industrial networks is by exploiting the “Modicon Communication Bus (Modbus)” and its variants, which are widely used in the oil and gas industry, particularly for pipeline operations. Cybercriminals can gain access to remote offshore operations through Modbus, which operates on a master–slave or server–client basis. Due to its lack of authentication and encryption in the “Modbus TCP protocol,” it remains highly vulnerable to cyberattacks (Mohammed et al., 2023).

ICS and IoT often have inherent security vulnerabilities that malicious actors can exploit. This section covers common attack types that could compromise these industrial systems (Mohammed, 2024; Stergiopoulos et al., 2020; Perdomo and Serdyuk, 2021; Bundi and Mayieka, 2020):

1. *Malware*: The most common attack against ICS, malware includes viruses, trojans, and other malicious programs designed to damage or disrupt systems.
2. *Ransomware*: A high-profile malware attack that locks and encrypts critical data, files, or systems, preventing access until a ransom is paid (Vaughn, 2025; CISA, 2020; Vejlgaard Sørensen, 2023).
3. *Man-in-the-Middle (MITM) attacks*: These attacks intercept and alter communications, potentially leading to data leakage, unauthorized control of PLCs, or the manipulation of actuators to change operational states (e.g., closing valves or adjusting sensor temperature thresholds; Zhang et al., 2019).
4. *Denial of Service (DoS) attacks*: Attackers exploit network sniffing techniques to analyze traffic and craft malicious packets that flood the network, rendering process control requests ineffective.
5. *Injection attacks*: Cybercriminals can compromise engineering workstations in control centers to manipulate legitimate commands, causing pumps, actuators, or other ICS components to behave improperly, potentially leading to catastrophic system failures.
6. *Phishing attacks*: Social engineering tactics deceive users into revealing sensitive information or credentials, which attackers use to compromise IoT devices or ICS systems. Studies show that 43% of cyberattacks result from a lack of end-user awareness, making phishing a significant attack vector (Bundi and Mayieka, 2020).

Over the years, ICS and IoT have witnessed significant evolution in both the sophistication of attacks and their potential consequences. Table 1 provides an overview of historical threats, vulnerabilities, and attacks on ICS and IoT, highlighting key incidents and their impact (Hemsley and Fisher, 2018; Mehdiyev and Hashimovv, 2024).

**Table 1:** ICS and IoT cyber incidents.

No	Year	Type	Name	Desecration
1	2000	Attack	Maroochy Water	The “Maroochy” experienced system failures due to a cyberattack that caused the release of more than 265,000 gallons of untreated sewage.
2	2010	Malware	Night Dragon	Attackers used sophisticated malware to target global oil, energy, and petrochemical companies using remote access tools to gain control of computer systems and collect information by compromising ICS and IoT.
3	2012	Campaign	Gas Pipeline Cyber Intrusion	ICS-CERT identified an active series of sophisticated cyber-intrusions targeting the natural gas pipeline sector involving spear-phishing attacks.
4	2012	Malware	Shamoon	Attacked the world’s largest oil producer in Saudi Arabia and the second-largest producer of liquid natural gas in Qatar. These were hit by similar malware (Almaiah and Almomani, 2020).
5	2014	Malware	Black Energy	Malware that targeted human–machine interfaces (HMIs) in ICSs.
6	2014	Attack	German Steel Mill	Attack on an unspecified German steel mill. The attackers used advanced tactics like “spear-phishing” to gain access to the business and production networks, causing multiple control system failures.
7	2016	Malware	Return of Shamoon	Thousands of computers in Saudi Arabia’s civil aviation agency and other Gulf State organizations were wiped. The second Shamoon targeted critical infrastructure.
8	2016	Attack	Ukraine Power Grid Attack No. 2	Ukraine experienced another major cyberattack on its power grid. Cyberattackers tripped breakers in 30 substations, turning off electricity to “225,000” customers by manipulating “SCADA” systems.
9	2016	Attack	Kemuri Water Company	Attackers gained access to hundreds of the PLCs used to manipulate control applications and altered water treatment chemicals.
10	2017	Attack	TRITON/Trisis/HatMan	This attack was designed to disrupt critical infrastructure by targeting the safety instrumented systems of electric products. This malware represented a concern for its capability to target industrial safety systems in a way that could potentially cause physical damage or harm.
11	2021	Attack	DarkSide Attack on Colonial Pipeline	Colonial Pipeline suffered a ransomware attack conducted by the DarkSide hacking group. The attack forced the largest oil pipeline operator in the US to halt all operations (Beerman et al., 2023).

The analysis was conducted using various cybersecurity resources, including research studies and published reports. The focus is not merely to list cyberattacks but to highlight significant cyber threats that have impacted ICS, IoT devices, and critical infrastructure over the years amid ongoing technological advancements. While many cyber incidents were not explicitly mentioned, their occurrence worldwide underscores the rapid evolution of threat actors’ technical capabilities. These breaches can result in production losses, increased health, safety, and environmental risks, as well as severe reputational damage. Therefore, cybersecurity must be a priority, and developing a robust attack detection system for early-stage threat identification is a crucial step in securing vast and critical sectors such as oil and gas.

## 3 Literature review (related works)

This review aims to explore and examine the existing research and literature surrounding the real-time detection of cyberattacks on industrial control systems (ICS) and the Internet of Things (IoT). The objective is to identify common themes, evaluate the strengths and weaknesses of previous studies, and highlight gaps or unresolved issues. This section is organized into parts that provide an overview of foundational theories and concepts, highlight employed methodologies, discuss key findings, and analyze gaps and limitations identified in existing literature.

### 3.1 Phishing attack detection

Abedin et al. (2020) utilized publicly available datasets from Kaggle containing 32 attributes and 11,504 instances, including both phishing and legitimate website data. Three supervised machine learning (ML) algorithms—K-nearest neighbor (KNN), Logistic Regression (LR), and Random Forest Classifier—were used for classification. RFC achieved the highest precision (97%) and recall (99%), outperforming KNN and LR. However, the study focused solely on URL-based phishing detection without considering other types of phishing attacks.

Pithawala et al. (2021) used a dataset of 1,780 entries with 19 features extracted from verified sources of phishing URLs. ML classifiers, including naïve Bayes (NB), LR, and RFC, were applied to detect phishing in short URLs. NB achieved the highest accuracy (99.4%). The study was limited in scope due to its small dataset size and focus on a single attack type.

### 3.2 ICS and IoT cyberattack detection

Ahmed and Tjortjis (2022) used the Ton-IoT dataset, which contains 461,043 samples with 43 features across six categories. ML models such as LR, Gaussian naive Bayes (GNB), DT, RFC, KNN, and Extreme Gradient Boosting (XGBoost) were applied. DT, RFC, KNN, and XGBoost outperformed LR and GNB, achieving over 99% accuracy. However, multi-class attack classification was not explored.

An “ICS cyber test kit” (Mubarak et al., 2021) was developed to generate industrial network traffic data for various attack scenarios. The dataset included Modbus/TCP, Ethernet/IP, and IEC 61850 network traffic. Ensemble ML techniques, including deep learning (DL) models (RNN with LSTM), were utilized. The ensemble approach achieved a prediction accuracy of 99.91%. However, computational complexity and real-time deployment challenges were not addressed.

The Microsoft Malware Prediction dataset, containing 4,000 entries and 64 features, was used. DT algorithm variants (C4.5 and C5.0; Yeboah-Ofori, 2020) were applied to predict malware infections. The DT model effectively detected cyberattacks but was not compared with other ML models, and the dataset size was relatively small.

### 3.3 DDoS attack detection

The authors of this work applied the AdaBoost algorithm (Syafiuddin et al., 2023) to detect SYN flood and UDP lag attacks using the CICDDoS2019 dataset. AdaBoost outperformed other ML models but lacked a detailed analysis of feature selection and hyperparameter tuning.

There has also been an analysis of Modbus (Saharkhizan et al., 2020) network traffic with five attack types (MITM, Ping DDoS, Query Flood, TCP SYN, Helvetica Neue Flood). An ensemble model combining multiple LSTM architectures with a Decision Tree achieved over 99% accuracy. However, the study was limited to the Modbus protocol, and computational efficiency for resource-constrained IoT devices was not evaluated.

Research in Alasmari et al. (2023) used the CI-CDDoS2019 dataset (400,000 datapoints) and proposed a CNN-LSTM model for DDoS detection. The CNN-LSTM model outperformed other ML models, achieving 99.51% accuracy. However, preprocessing steps and computational complexity were not thoroughly analyzed.

### 3.4 Multi-attack detection

Works in Canadian Institute of Cybersecurity (2017) utilized the CICIDS2017 dataset, which contains 183,910 instances across multiple attack types. Supervised ML models (DT, RFC, AdaBoost, KNN, SVM) were applied, with DT achieving the highest accuracy (99.84%). The study focused exclusively on port-scanning attack detection without extending to other attack types.

Works in Huynh et al. (2023) used real-time data captured from an ESP32 microcontroller and the CICIoT2023 dataset for DoS and DDoS attack detection. SVM and LR models achieved 99% accuracy. However, the dataset was limited to a single attack type, and feature engineering was minimal. Table 2 provides a summary of the reviewed literature, highlighting key datasets, models, and findings.

**Table 2:** Summary of previous works on detection of ICS and IoT cyber attacks.

Work	Objective	Dataset	Algorithm	Limitation of the study
Abedin et al. (2020)	Phishing attack detection	32 attributes and 11,504 instances. The dataset contains both phishing and legitimate website data.	Three supervised ML algorithms: KNN, LR, and RFC.	- Use of a small dataset - Binary classification (two class)
Pithawala et al. (2021)	Short uniform resource locators	Dataset 1780 entries with 19 features related to phishing and non-phishing URLs	Three ML algorithms: naive Bayes (NB), Logistic Regression (LR), and Random Forest Classifier (RFC)	- Use of a small dataset - Binary classification (two classes)
Ahmed and Tjortjis (2022)	IoT-Botnet attack detection using real-time heterogenous data	Dataset: 461,043 samples, with 65.07% normal traffic and 34.93% malicious traffic. The dataset consists of 43 features across 6 categories: connection activity, DNS, SSL, statistical, HTTP, and violation activity	Data preprocessing, feature engineering, and performance of several supervised ML algorithms: LR, GNB, DT, RF, KNN, and XGB. DT, RFC, KNN, and XGB outperformed LR and GNB, with an accuracy over 99% and F1-scores of 0.98–0.99	Binary classification (two classes)
Mubarak et al. (2021)	ICS cyber attack detection using cyber-kit datasets	Dataset: Network traffic data from different ICS protocols, such as Modbus/TCP, Ethernet/IP, and IEC 61850, along with a normal baseline and diverse industrial hacking scenarios. Deep packet inspection (DPI) was used to extract metadata features from network traffic data and the final dataset matrix is (30,608,16).	Ensemble ML including both traditional (LR, KNN, NB, RFC, ANN, SVM, DT) and DL (RNN, LSTM). The ensemble approach resulted in 99.91% prediction	Complexity and diversity of ICS not captured - Binary classification as secure and insecure - No details about model performance
Yeboah-Ofori (2020)	Classification of malware attacks	Microsoft Malware Prediction dataset: 4000 entries with 64 columns representing various metadata about the machines and malware infections	The C4.5 and C5.0 variants of the DT algorithm Accuracy = 83%	- One ML algorithm - Use of a small dataset - Binary Classification (two classes)
Syafiuddin et al. (2023)	Detection Syn flood and UDP lag attacks based on AdaBoost	CICDDoS2019 dataset, which is a dataset of network traffic containing simulated DDoS attacks on 25 different network users	AdaBoost algorithm outperformed with other machine learning algorithms (RFC, Simple Logistic, and REP Tree.) with values above 47.2% for detecting SYN flood and UDP lag attacks	No details about feature selection and hyperparameter tuning Binary classification only
Saharkhizan et al. (2020)	Ensemble of Deep RNN for IoT cyber attacks	Dataset: Modbus network traffic, including 5 types of attack (man-in-the-middle attack, Ping, DDoS Flood attack, Modbus Query Flood attack, and TCP SYN DDoS Flood attack) The dataset was captured in pcap files and pre-processed to extract 83 features totaling 5,859,085 samples	Integration of LSTM models into an ensemble model. Then aggregate output using a DT. Ensemble of LSTM accuracy = 99% for a window size of 40 packets	Evaluated only for Modbus protocol traffic for cyberattack detection - Computational overhead of DL is a concern for IoT devices
Alasmari et al. (2023)	CNN-LSTM based approach for DDoS detection	CI-CDDoS2019 dataset contains network traffic data with 400,000 datapoints and 12 different types of DDoS attacks as well as benign traffic	CNN-LSTM model has been used and achieved an accuracy of 99.51% in detecting DDoS attacks, outperforming the other ML algorithms tested	No information or details about the preprocessing steps that have been done on the data. No details about the computational complexity of the CNN-LSTM model. Binary classification as Benign and DDoS
Canadian Institute of Cybersecurity (2017)	Port-scanning attack detection	CICIDS2017 dataset: Network attacks with 62 columns and 183,910 instances. Includes network traffic in packet-based and bidirectional flow-based format	Five ML algorithms: DT, RFC, AdaBoost, KNN, and SVM.	The study focused only on detecting the Port-Scanning attack as 0 and 1. Evaluated the performance of the algorithms on other types of attacks present in the CICIDS2017 dataset
Huynh et al. (2023)	DOS attack detection	Two datasets - a real-time dataset captured using a packet sniffer on an ESP32 microcontroller, and the CICIoT2023 dataset which contains a wider variety of DoS and DDoS attacks.	Two algorithms: SVM and LR - for binary classification. Both SVM and LR accuracy = 99%	Real-time dataset contained only a single type of DoS attack, which limits the generalizability of the models. Feature engineering was limited to just two features (frame length and packet inter-arrival time) for the real-time data and seven features for the CICIoT2023 dataset. Binary classification was only applied for analysis.

Despite advancements, several challenges remain in applying ML and DL techniques for ICS and IoT cybersecurity. While these models effectively detect and classify cyberattacks by learning patterns and extracting meaningful features, existing studies exhibit key limitations. Many focus on binary classification, restricting their ability to detect diverse and evolving threats. Additionally, reliance on limited datasets fails to capture the complexity of real-world cyber threats. Gaps in data preprocessing, feature selection, and optimization further hinder performance. Although deep learning ensembles enhance classification accuracy, they introduce significant computational overhead, posing challenges for real-time deployment in resource-constrained environments. Addressing these limitations is crucial for developing scalable, efficient, and adaptive cybersecurity solutions for critical infrastructure.

## 4 Necessity of multi-class cyberattack detection

Enhancing cyberattack detection in ICS and IIoT environments requires more effective classification models capable of multi-class classification. This study addresses existing limitations by introducing a feature engineering approach that encodes critical attributes such as URLs and IP addresses, which are essential for accurate classification. Additionally, key dataset attributes—including http.request.method, http.referer, http.request.version, dns.qry.name.len, mqtt.conack.flags, mqtt.protoname, and mqtt.topic—are categorized to improve classification performance.

Beyond feature engineering, feature selection techniques are employed to identify the most relevant attributes, optimizing model performance for detecting malicious behavior patterns in ICS and IIoT datasets. This paper categorizes cyberattacks into five distinct groups, each with unique characteristics and impacts, emphasizing the need for tailored detection strategies. A robust multi-class classification model strengthens industrial security by accurately identifying and mitigating diverse threats. Table 3 summarizes the varying impacts of each attack type and their detection priority.

**Table 3:** Attack categorization.

Attack category	Description	Potential impact	Priority for detection
DDoS (Distributed Denial of Service)	Overloading system resources to cause disruption	Service outages, loss of availability, disruption of ICS operations	High
Information Gathering	Unauthorized data collection from systems	Leakage of sensitive information, intelligence gathering by attackers	Medium
MITM (Man-in-the-Middle)	Intercepting communication between systems	Data tampering, unauthorized control of ICS operations	High
Injection	Inserting malicious code or data into systems	System malfunction, compromise of system integrity	High
Malware	Software designed to damage or disrupt systems	Data theft, system corruption, operational failure	Critical

This study presents optimized stacked ensemble models (Stacked Ensemble_1 and Stacked Ensemble_2) for classifying 15 distinct types of malicious behavior in ICS and IIoT environments. Designed to enhance accuracy while minimizing computational costs, these models are evaluated against other optimized approaches, including bagging, boosting, and individual classifiers. A comprehensive analysis further demonstrates the improvements achieved by the proposed methodology compared to previous studies.

## 5 Methodology implementation

### 5.1 Dataset description

The Edge-IIoT dataset is a comprehensive and realistic cybersecurity resource for IoT and IIoT applications, publicly available through IEEE. Generated using a purpose-built IoT/IIoT testbed, it encompasses a large and representative set of devices, sensors, protocols, and cloud/edge configurations from over 10 types of IoT devices. The dataset identifies and analyzes 14 attacks targeting IoT and IIoT connectivity protocols, categorized into five threat types: DoS/DDoS attacks, Information Gathering, Man-in-the-Middle attacks, Injection attacks, and Malware attacks (Ferrag et al., 2022). It is ranked in the top 1% of datasets in the Web of Science.

**Figure 2:**
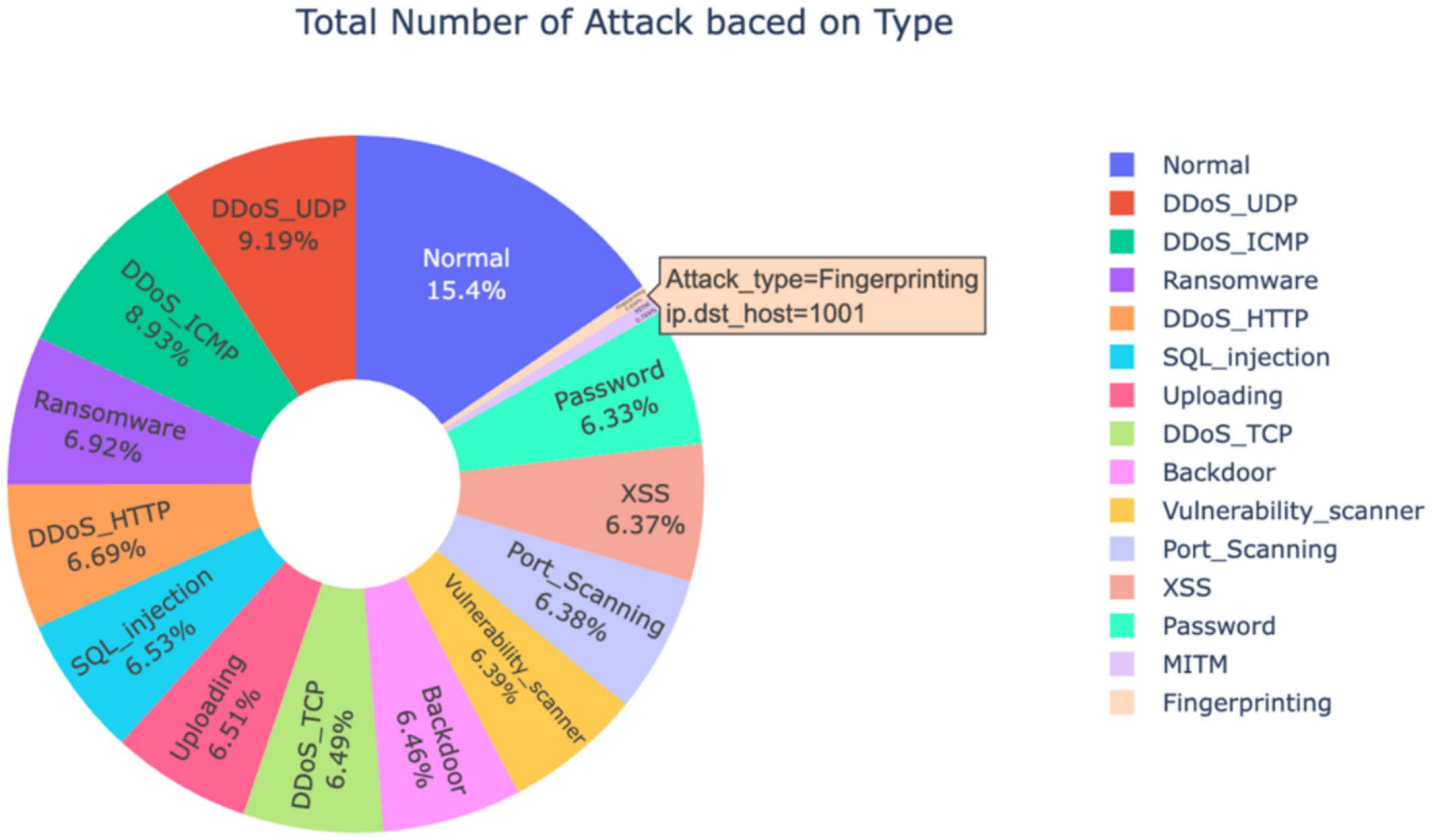
Example edge-IIoTset features.

The dataset consists of 63 columns capturing various data types relevant to IIoT and IoT systems, as well as potential attack scenarios. The distribution of 157,800 samples across 15 attack types is shown in Table 4 and Figure 2. Prior to data analysis for classification, specific preprocessing steps are necessary to prepare the dataset for model training. These steps involve cleaning, transforming, and organizing the data to ensure it is suitable for modeling. The preprocessing approach depends on the data characteristics and modeling objectives. The goal is to align the data with the model and ensure precise outcomes. This includes actions such as rescaling frames to improve small object detection and managing redundant frames to prevent overfitting and enhance accuracy. To maintain data quality, essential cleaning steps are first performed, including handling missing values, removing duplicates, standardizing formats, and resolving other data quality issues. These procedures ensure that the data is accurate, complete, and consistent, making it reliable for analysis and decision-making.

**Table 4:** Category of threats in edge-IIoTset.

DoS/DDoS	Information gathering	MITM	Injection	Malware
TCP SYN Flood DDoS attack = 10,247	Port Scanning = 10,071	ARP Spoofing attack + DNS Spoofing attack = 1,214	XSS = 10,052	Backdoor attack = 10,195
UDP flood DDoS attack = 14,498	OS Fingerprinting = 1,001		SQL injection = 10,311	Password cracking attack = 9,989
HTTP flood DDoS attack = 10,561	Vulnerability scanning attack = 10,076		Uploading attacks = 10,269	Ransomware attack = 10,925
ICMP flood DDoS attack = 14,090				
Normal	24,301	Total	157,800	

### 5.2 Feature engineering

One of the columns in the Edge-IIoTset dataset, ‘frame.time,’ indicates the arrival time of data. Initially, the data type of this column was an object, so it was converted to a datetime data type. This conversion ensures that the ‘frame.time’ column contains valid and consistent datetime values, which is essential for subsequent data analysis and processing. Table 5 illustrates the ‘frame.time’ column before and after feature engineering.

**Table 5:** Fields before and after feature engineering.

Feature engineering process	Feature name	Before feature engineering	After feature engineering
Change data type	“Frame.time”	Object	Datetime 64
Datetime conversions	“Frame.time”	2021 23:24:32.698981000	2021-01-01 23:24:32.698981
Integer conversion	“IP address”	192.168.0.128	3,232,235,648
Categorical conversions	‘http.request.method’, ‘http.referer’, ‘http.request.version’, ‘dns.qry.name.len’, ‘mqtt.conack.flags’, ‘mqtt.protoname’, and ‘mqtt.topic’	False or True	0 or 1
Missing values in the records	All Features	(157,800, 63)	(142,095, 63)

The dataset contains four columns representing IP addresses: ‘ip.dst_host,’ ‘ip.src_host,’ ‘arp.dst.proto_ipv4,’ and ‘arp.src.proto_ipv4,’ all initially having an object data type. These need to be converted into integers for the model to process them effectively. To achieve this, we created a function called ip_to_int(ip) that takes an IPv4 address as a string and converts it to its corresponding integer representation. This conversion facilitates tasks such as sorting or performing numerical comparisons on IP addresses.

The function uses the built-in ipaddress. IPv4Address(ip) from Python’s ipaddress module to create an IPv4Address object, which is then converted to an integer using the int.() function. If the input is not a valid IPv4 address (e.g., addresses like “0.0” or “0”), the ValueError exception is caught in the except block, and the function returns 0. This approach ensures a consistent and predictable output for invalid IP addresses. Table 5 illustrates the IP addresses before and after feature engineering.

Next, is the encoding of the URLs in our dataset. The ‘http.file_data’ column consists of different values extracted from the web. To preprocess this data, we define a function called extract_text (html) that takes an HTML string as input and extracts the text content from it. The function creates a BeautifulSoup object from the input HTML using the html.parser, and then extracts the text content using the soup.get_text() method. Once we have extracted the text, it is stored in a variable called text_content. From this text_content variable, we then extract two new numerical features: text_length (the length of the text content) and word_count (the number of words in the text content). We convert these two new features to the float data type. Finally, we scale the numerical features (‘text_length’ and ‘word_count’) using the MinMaxScaler from the sklearn.preprocessing module. This scales the features to the range of [0, 1], which can be useful for machine learning algorithms.

There are some columns in this dataset that can be categorized, such as (‘http.request.method’, ‘http.referer’, ‘http.request.version’, ‘dns.qry.name.len’, ‘mqtt.conack.flags’, ‘mqtt.protoname’, and ‘mqtt.topic’). We define a function called encode_text_dummy (df, name) that performs one-hot encoding on a text column in each DataFrame. This is a preprocessing step for ML models that cannot directly handle categorical text data. The one-hot encoding creates a binary column for each unique value in the specified column, where a value of True indicates the presence of that value and False indicates its absence. This transformation allows the model to work with the text data in a numerical format. Table 5 shows the categorical conversion before and after preprocessing.

For the target column that we are going to predict, we used the LabelEncoder from the sklearn.preprocessing module to encode the ‘Attack_type’ column in the DataFrame. The LabelEncoder is a useful tool for converting categorical variables into a numerical format, which is often required for many ML algorithms. Most ML algorithms require numerical inputs, and the LabelEncoder provides a straightforward way to convert categorical variables into a format that the model can understand. After applying the encoding, the ‘Attack_type’ column in the DataFrame contains numerical values instead of the original categorical values. The model can now treat these values as numerical data and use them in the training and prediction processes. Table 6 shows the target column before and after label encoding.

**Table 6:** Target column before and after label encoding.

“Attack type” before label encoding		“Attack type” after label encoding	
Backdoor	10,195	0	10,195
DDoS_HTTP	10,561	1	10,561
DDoS_ICMP	14,090	2	14,090
DDoS_TCP	10,247	3	10,247
DDoS_UDP	14,498	4	14,498
Fingerprinting	1,001	5	1,001
MITM	1,214	6	1,214
Normal	24,301	7	24,301
Password	9,989	8	9,989
Port_Scanning	10,071	9	10,071
Ransomware	10,925	10	10,925
SQL_injection	10,311	11	10,311
Uploading	10,269	12	10,269
Vulnerability_scanner	10,076	13	10,076
XSS	10,052	14	10,052

### 5.3 Feature selection

After performing all the preprocessing steps, the initial number of features increased to 94 columns. We know that the more features you have, the volume of the feature space grows exponentially. This makes it increasingly difficult for the model to find patterns and relationships in the data. With high-dimensional data, the model may struggle to generalize well, which can lead to overfitting and poor performance on new, unseen data. Additionally, training machine learning models on datasets with many features can be computationally expensive and time-consuming.

To address these issues, we dropped unnecessary columns and shuffled the data in random order. Shuffling the data can be useful for training models to ensure that the training and test sets are representative of the overall data distribution. These steps reduced the number of features to 75. Next, we performed feature selection using the Variance Threshold method to remove constant or near-constant features from the dataset, which helps ensure the reliability of the data. The reason for choosing the variance threshold is that it is computationally the simplest and fastest method. It evaluates each feature based solely on its variance and eliminates those with low variance, making it a quick preprocessing step. This variance threshold does not require training or model fitting and works directly with the dataset, making it much faster in terms of execution compared to other methods like Recursive Feature Elimination. Additionally, the Variance Threshold is very fast and requires minimal tuning, making it suitable for large datasets, which fits our Edge-IIoT dataset well.

We started by separating the dataset into features (X) and the target variable (Y). The X variable contains all the columns except the “Attack_type” column, which is assigned to Y. The threshold parameter was set to 0.00001, meaning that any feature with a variance less than or equal to this value would be considered constant and removed. A list of column names identified as constant or near-constant by the variance threshold object was then dropped from the dataset, as shown in Table 7.

**Table 7:** Number of features.

Number of features				
Original	After URL-encoded data	After categorical conversions	After dropping	Feature selection
63	66	94	75	67

On the other hand, the number of features increased during the preprocessing steps, based on some previous studies that suggest increasing the number of features by simplifying the value of columns to Boolean-like categorical sections can positively impact models. This approach can reduce the sparsity of the data, which is particularly helpful when dealing with high-dimensional, sparse data, as it can make it easier for the model to find patterns and relationships. Additionally, Boolean features are generally more straightforward to interpret than complex numerical features, leading to improved interpretability. Furthermore, Boolean features are less computationally intensive to work with compared to high-cardinality features, resulting in faster training and inference times (Singh, 2021).

### 5.4 Data training

In this study, the dataset was split into training and test sets using an 80/20 split (80% for training, 20% for testing). This ensures that the model is trained on a sufficiently large portion of the data while still having a held-out test set to assess its generalization capabilities. The random_state parameter is set to 111 to ensure that the split is reproducible, which is important for model evaluation, hyperparameter tuning, and model selection.

Additionally, we applied the “Synthetic Minority Over-sampling Technique (SMOTE)” to address the issue of class imbalance in the training data after splitting the dataset. This means that the training portion was oversampled using SMOTE to handle class imbalance, while the validation and test sets remained untouched. Applying SMOTE in this way prevents data leakage and ensures that the model is evaluated on unseen data, providing a fair and reliable estimate of its performance. Class imbalance is a common problem where one class (the majority class) is significantly more prevalent than the other classes. This can cause models to be biased toward the majority class and perform poorly in the minority class. The SMOTE algorithm is a powerful technique for addressing class imbalance. It works by generating synthetic samples of the minority class, effectively increasing the number of instances of the minority class in the training data. This helps to balance the class distribution and improves the model’s ability to learn from the minority class (Satpathy, 2024; Widodo et al., 2024; Alex, 2025).

## 6 Proposed methodology

The primary objective of this research is to develop an optimized and efficient machine learning model to detect the malicious behavior of ICS and IoT. To achieve this, a stacked ensemble approach has been chosen for developing a high-quality machine learning model that effectively produces predictive results.

The motivation behind developing stacked ensembles is that they can enhance the accuracy of various machine learning models. In addition, the stacked ensemble approach offers numerous advantages in improving predictive performance, model diversity, flexibility, and interpretability such diversity in stacking can also mitigate the problem of overfitting, making stacked ensemble models more robust for different types of data. The primary reason for opting for stacking-based ensembling is that it does not rely on existing methods like bagging or boosting but allows meta models to learn the bias patterns of the base models and make adjustments to enable the meta model to make final predictions using additional data.

The primary focus of this work is not on proposing a novel algorithmic architecture but rather on designing and validating an effective, domain-informed ensemble framework tailored for cybersecurity intrusion detection. Our approach strategically integrates well-established machine learning models—XGBoost, LightGBM, Extra Trees Classifier, and Logistic Regression—within a stacking ensemble to leverage their complementary strengths. For instance, the inclusion of Logistic Regression adds an interpretable component that provides insights into decision-making processes, aiding cybersecurity analysts in understanding attack behaviors. Furthermore, our work demonstrates significant empirical improvements in attack detection accuracy, robustness, and interpretability, which are particularly important for practical cybersecurity deployment. The intention is to show how a carefully engineered combination of existing models can significantly enhance performance, robustness, and interpretability when applied to complex, imbalanced security datasets.

Based on the motivation for the stacked ensemble approach, two stacked ensemble models were developed. The Stacked Ensemble_1 model is a standard approach that was constructed using the Boosting algorithm as the base learner, which improves misclassification, making it a strong learner. The output from these boosting algorithms is handled by a Random Forest Classifier, which serves as a meta-learner based on a subset of data and features derived from the prediction output of the Boosting algorithms (AdaBoost and XGBoost). This ultimately enables the model to learn the bias patterns of the boosting algorithms and produce the final prediction through the Random Forest Classifier, making it a highly effective and predictive model (Alsolami et al., 2024).

An improvisation over Stacked Ensemble_1 is Stacked Ensemble_2, which has been developed with multiple base models and an RFC as the final estimator to improve generalization. Combining the predictions of these models helps to reduce error, as each base model makes errors in different areas, captures different patterns, and structures the dataset in various ways. Stacked Ensemble_2 includes a variety of base models, each with different learning mechanisms:

- Logistic Regression (linear model)
- XGBoost (boosting model)
- LightGBM (gradient boosting model)
- Extra Tree (ensemble of decision trees)

The primary goal of this research is to build a Stacked Ensemble_2 with different decision boundaries (linear and non-linear) to increase the overall diversity of the model, which is key to improving performance. If all base models in the ensemble were non-linear or tree-based models, they might make similar errors, and the ensemble would not be as effective at correcting each other’s weaknesses. Using a complex model like XGBoost, LGBM, or Extra Tree Classifier, along with a simpler model like LR, will make the final decision process more interpretable for the ensemble model. This approach will enable Stacked Ensemble_2 to access a wider range of decision-making strategies. LR is included as a basic interpretable classifier to establish a benchmark or baseline. The final estimator, RFC, will combine predictions from both simple and complex models to improve robustness and reduce the likelihood of overfitting, demonstrating how well this approach using a simple linear model performs before adding complexity.

LightGBM contributes efficient gradient boosting and fast convergence, Extra Trees provides strong variance reduction through randomized tree averaging, and Logistic Regression adds interpretability by offering linear decision boundaries that are more transparent to analysts. This methodological design allows the ensemble to maintain high predictive performance while ensuring explainability and operational feasibility. Thus, the contribution of this study lies in the systematic integration, optimization, and evaluation of these models for real-world ICS and IoT security applications, bridging the gap between theoretical model design and deployable cybersecurity solutions.

The rationale for selecting Stacked Ensemble_2 over Stacked Ensemble_1 is based on its broader capacity to address the complexities inherent in ICS and IoT security datasets. While Stacked Ensemble_1 combines boosting models (XGBoost and AdaBoost) with an RFC to leverage bias reduction and variance stabilization, Stacked Ensemble_2 incorporates a more diverse set of base models —XGBoost, LightGBM, Extra Trees, and Logistic Regression, with RFC as the meta-model.

Stacked Ensemble_2 offers several advantages, including LightGBM, which is highly effective at capturing complex attack patterns, making it well-suited for identifying sophisticated threats. The bagging model (Extra Trees) provides robustness against noisy and imbalanced data, common in security datasets with few attack instances. Logistic Regression adds interpretability, enabling a better understanding of the decision-making process.

The inclusion of LightGBM and Extra Trees offers fast inference times, facilitating real-time threat detection. Overall, Ensemble_2’s heterogeneity and tailored components make it more effective in handling the challenges of ICS and IoT security environments compared to Stacked Ensemble_1.

For both stacking configurations, a k-fold cross-validation strategy (with k = 5) was applied to generate out-of-fold (OOF) predictions from each base model. These OOF predictions were then used as input features for the meta-learner, ensuring that the meta-learner was trained exclusively on data unseen by the base models during training. The stack_method = ‘predict_proba’ parameter was used so that class probability estimates and discrete class labels were passed to the meta-learner, allowing it to learn finer decision boundaries and improve overall calibration.

The stacking procedure was repeated under a 5 × 3 repeated stratified cross-validation framework. This provided reliable mean and standard deviation estimates for all performance metrics and ensured consistent evaluation across different data partitions. All experiments were conducted on a MacBook Pro (Apple M1, 8 GB RAM) using an Anaconda-managed environment.

Through this design, both stacked ensemble models maintained strict separation between training and meta-training data, thereby preventing data leakage. The resulting performance metrics thus reflect true generalization ability rather than overfitting effects caused by shared training data between stages.

**Figure 3:**
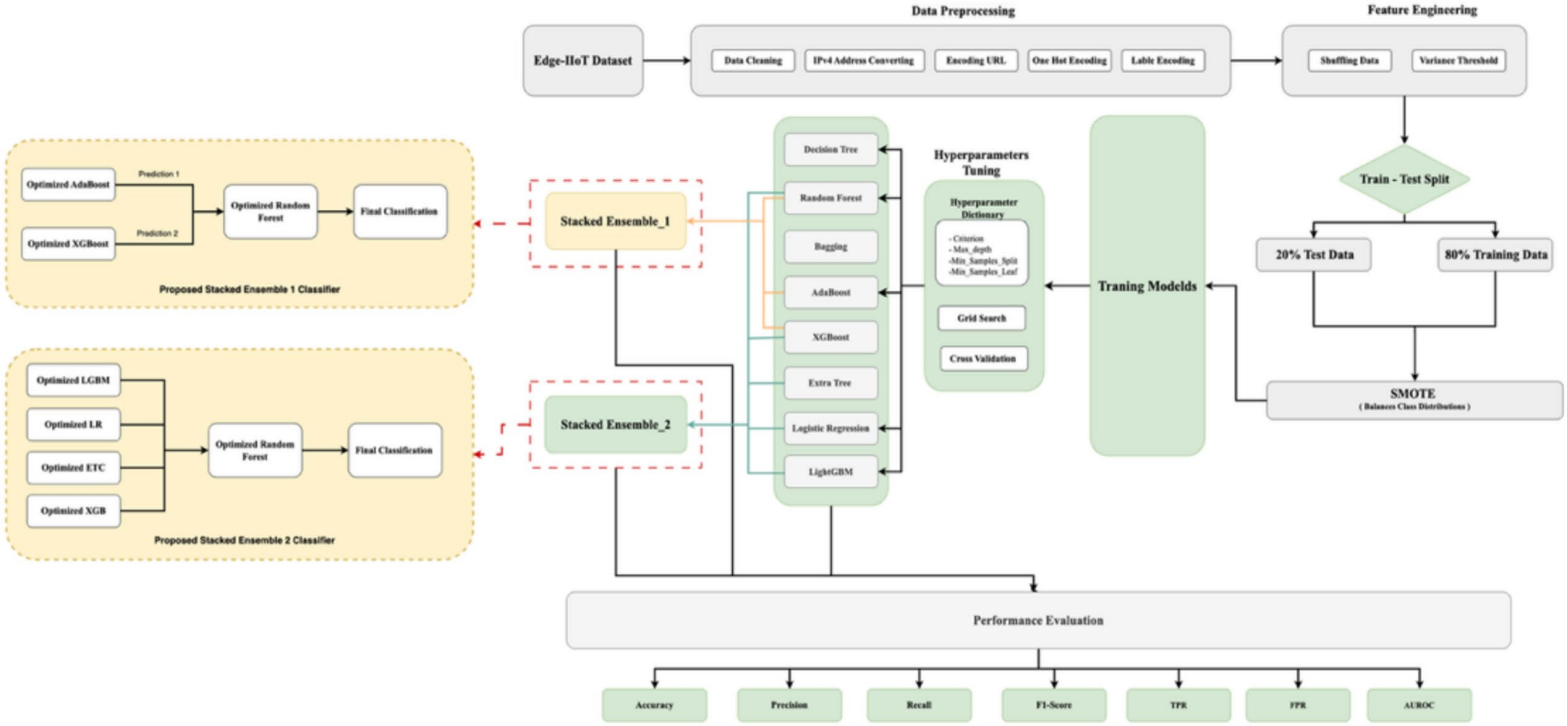
Proposed high-level architecture for cyberattack classification.

The stacked ensemble models developed are optimized and compared against different ensemble machine learning models, such as Bagging, Boosting, Extra Trees, and individual classifiers like Logistic Regression and decision tree, to validate the best model for detecting malicious behavior in ICS and IoT. The study aims to identify the approach for accurately classifying malicious instances within the dataset. Figure 3 shows the high-level architecture of cyberattack classification in IIOT using the optimized machine learning model. The scripts and datasets related to this experiment are uploaded to the GitHub repository (BATL, 2025).

### 6.1 Machine learning algorithms

#### 6.1.1 Logistic regression

Several advantages make Logistic Regression (LR) a good choice for this paper. The simplicity and interpretability of this model make it a great starting point for classification problems, where the coefficients from the trained model can be interpreted to understand the relationships between features and the target class. Additionally, in our paper, we are dealing with large, high-dimensional datasets, where LR is computationally efficient for handling a large number of features. The flexibility of this model enables us to adapt it for multi-class classification using strategies like the one-vs-rest (OvR) technique. In this model, multiple binary classifiers are trained, each for a separate class, allowing the model to handle more than two classes effectively, with the class having the highest probability chosen as the prediction. To find the best parameters for our LR, we started by defining a dictionary that specifies the hyperparameters to be tuned using GridSearch CV (Nashaat, 2023). The hyperparameters tuned to optimize the LR and their roles are shown in Table 8.

**Table 8:** Hyperparameter of logistic regression.

Hyperparameter	Description	Value tuned range
C	Controls the regularization strength, helping prevent overfitting, while a larger value of C allows the model to fit the training data more closely.	[0.01, 0.1, 1, 10]
Max Iter	Controls the maximum number of iterations the algorithm should run. It helps ensure the model converges.	[30, 40, 50]
Penalty	Regularization penalties such as ‘l1’ (Lasso regularization) or ‘l2’ (Ridge regularization). L1 penalty can produce sparse models, while L2 penalty generally produces models with smoother decision boundaries.	[“L1,” “L2”]

#### 6.1.2 Decision tree classifier

Several advantages make decision trees a good choice for this paper. They are robust to noise and can tolerate missing information, making them suitable for handling various types of attributes, including irrelevant and redundant ones. In addition, decision tree algorithms have a low computational cost, which makes them a practical choice. Like other ML models, we can also improve their performance through hyperparameter tuning due to the high number of possible configurations and their significant impact on predictive performance (Mantovani et al., 2024). To find the best parameters for our DT, we started by defining a dictionary that specifies the hyperparameters to be tuned. The hyperparameters tuned to optimize the decision tree are shown in Table 9.

**Table 9:** Hyperparameter of decision tree.

Hyperparameter	Description	Value tuned range
Criterion	Function to measure the quality of a split and find the best split when building a decision tree, either ‘gini’ or ‘entropy’.	Gini or entropy
Max depth	Controls model complexity. A higher max depth allows for more complex trees but can also lead to overfitting.	None, 15, 20, 25 (25)
Min samples split	Minimum number of samples required to split an internal node, which can help control model complexity.	4. 10. 20 (10)
Min samples leaf	Minimum number of samples required to be at a leaf node, which helps control overfitting.	2, 4, 8 (2)

### 6.2 Ensemble learning classifier

Combining multiple models or algorithms to improve the overall predictive performance of a system is a powerful machine learning technique called Ensemble Learning. The key goal of this ensemble approach is to leverage the complementary strengths of multiple base models, capture diverse patterns in ICS and IoT data, and produce more robust and accurate predictions of cyberattacks. The ensemble model aims to outperform individual base classifiers and provide a more reliable ICS and IoT attack detection system.

Subsequently, the prediction values would be used by ICS and IoT security for monitoring systems and detecting the presence of cyber threats. After a comprehensive study of existing models for ICS and IoT attack detection, considering their weaknesses identified in the reviewed literature, such as poor feature engineering, model overfitting, and inability to generalize to new attack types, the ensemble learning models in this study were designed to improve upon the existing systems with better accuracy. This section of the paper presents ensemble learning models for classifying harmful ICS and IoT attacks. Different types of ensemble learning techniques are discussed: (1) Random Forest Classifier, (2) Bagging, (3) Boosting, (4) Extra Trees, and (5) Stacked Ensemble.

#### 6.2.1 Random Forest classifier

The Random Forest model is a flexible learning model that can address a wide range of problems by creating multiple “decision trees” during the training period and producing an average forecast from all the decision trees involved. This model employs various “Exploratory Data Analysis (EDA)” methods and achieves a high accuracy score. One of its benefits is its ability to process large datasets with high complexity. It can analyze numerous input variables and identify the most significant ones, making it a useful dimensionality reduction model. In addition, the model highlights the importance of variables, which is a valuable feature when working with random datasets (Banerjee, 2023; Dash, 2023; Borah et al., 2020). To find the best parameters for our RF model, we started by defining a dictionary that specifies the hyperparameters to be tuned. The hyperparameters tuned to optimize the Random Forest are shown in Table 10.

**Table 10:** Hyperparameter of random forest.

Hyperparameter	Description	Value tuned range
Criterion	Function to measure the quality of a split and find the best split when building a decision tree, either ‘gini’ or ‘entropy’	Gini or Entropy (Entropy)
Max depth	Controls model complexity. A higher max depth allows for more complex trees but can also lead to overfitting.	None, 15, 20, 25 (None)
Min samples split	Minimum number of samples required to split an internal node; can help to control model complexity.	4. 10. 20 (4)
Min samples leaf	Minimum number of samples required to be at a leaf node, which will help to control overfitting.	2, 4, 8 (2)

The best hyperparameters found for the RFC model are: (1) the criterion ‘entropy’, which was better suited for the specific classification problem at hand, as it achieved higher predictive performance, and (2) Max_depth ‘None.’ This non-parametric approach can be beneficial for datasets with complex patterns, like Edge-IIoTset, that require deeper trees for accurate modeling. Allowing trees to grow without a depth limit provides the model with more flexibility to capture these intricate relationships. However, we need to be cautious about the risks associated with this approach, such as overfitting. Thus, parameters such as min_samples_split and min_samples_leaf play a crucial role in controlling the model’s complexity.

#### 6.2.2 Bagging (bootstrap aggregation)

Bootstrapping is a method that helps decrease the variance of the classifier and reduce overfitting by resampling data from the training set with the same cardinality as the original set (Akpan et al., 2023). In this paper, Bagging is composed of multiple DT Classifiers as the base estimators. Each Decision Tree classifier is trained on a different bootstrap sample, with the number of base estimators set to 500, the maximum depth of each DT Classifier set to 12, and the maximum number of training samples for each base estimator set to 300. The ensemble captures different patterns and perspectives in the data, and the aggregation of their predictions results in a more accurate and robust classification model. The max_depth parameter controls the complexity of the individual Decision Tree classifiers, while the max_samples parameter determines the size of the bootstrap samples used for training each base estimator. These hyperparameters were tuned to achieve the best performance for the ICS and IoT attacks.

#### 6.2.3 Boosting

Boosting is an iterative procedure in which each new model seeks to improve upon the errors of the previous models. The advantage of these algorithms is their adaptability to the strengths and weaknesses of the weak learners, focusing more on the samples that are difficult to classify correctly. There are several types of boosting algorithms; in this paper, we used some of the most well-known and commonly used types:

- *AdaBoost (Adaptive Boosting)*: AdaBoost is one of the earliest and most influential boosting algorithms, utilized for both regression and classification tasks. It operates through a process of iteratively training weak learners and adjusting the weights of the training samples based on the performance of preceding learners. This approach concentrates greater attention on the samples that were difficult to classify accurately in previous iterations. The objective of the boosting technique is to train subsequent learners using adjusted versions of the training data that have been updated based on the training effects of the preceding learner. In this way, it is possible to significantly reduce the deviation of the model’s forecasts from the actual values. Ultimately, the model’s final prediction is simply the weighted consensus of all the learners (Shabaan and Nemer, 2024).
- *XGBoost (Extreme Gradient Boosting)*: “XGBoost” utilizes decision trees that are constructed by starting at the root and recursively partitioning the data based on specific criteria. This process continues until the desired level of accuracy is achieved. The decision tree is trained to minimize the gradient of the loss function, which is calculated in each iteration. To prevent overfitting and speed up the learning process, the objective function is normalized. XGBoost is recognized for its ability to model non-linear relationships between variables and its exceptional classification capabilities. Consequently, numerous researchers have highlighted the potential of machine learning in forecasting time series data (Bikmukhametov and Jäschk, 2021; Tissaoui et al., 2022). Table 11 shows the hyperparameter settings for the three boosting algorithms.
- Light Gradient Boosting (LGB): LightGBM is highly efficient in both time and space. It employs histogram-based algorithms that accelerate training by reducing the amount of computation required. These optimizations make LGB faster and more memory-efficient compared to other gradient boosting algorithms like XGBoost, particularly when handling large datasets. To find the best parameters for our LGBM, we started by defining a dictionary that specifies the hyperparameters to be tuned using GridSearchCV (Verma and Yadav, 2024). For this model, the tuned hyperparameters and their roles are shown in Table 12.

**Table 11:** Hyperparameters of boosting algorithms.

Hyperparameters	AdaBoost classifier	Extreme gradient boosting (XGBoost)
Parameters	Random state = 1	Random state = 1 Learning rate = 0.01

**Table 12:** Hyperparameters of light gradient boosting algorithm.

Hyperparameter	Description	Value tuned range
Num leaves	The number of leaves in each tree.	[31, 50, 70]
Learning rate	Controls the size of the step the model takes during each boosting iteration.	[0.01, 0.1, 0.2]
N estimators	The number of boosting rounds or trees to build.	[30, 40, 50]
Max depth	The maximum depth of each tree. Setting it to −1 means no depth limit.	[−1, 5, 10]
Subsample	The fraction of data used to train each individual tree. It can ensure that trees are not too similar to each other.	[0.6, 0.8, 1.0]
Colsample bytree	The fraction of features to consider when building each tree.	[0.6, 0.8, 1.0]

#### 6.2.4 Extra tree classifier

The Extra Tree Classifier constructs an ensemble of unpruned decision or regression trees using the classical top-down procedure. Two main distinctions between this method and other tree-based ensemble techniques are that it randomly chooses cut points when splitting nodes and utilizes the entire learning sample to grow the trees, rather than a bootstrap replica (Baldini, 2024; Geurts et al., 2006; Dhingra et al., 2023). The main concept and assumption behind the Extra Tree Classifier is a variant of the Random Forest algorithm, which aims to improve the performance and robustness of the ensemble by introducing additional randomness into the tree construction process. Unlike Random Forest, which selects the best split among a random subset of features, Extra Trees selects the split randomly from all available features. The Extra Tree Classifier algorithm is based on three fundamental hyperparameters:

- The number of decision trees in the ensemble, set to n_estimators = 1,000
- The number of features to be selected randomly, set to random_state = 42
- The maximum number of instances (features) to consider when looking for the best split, set to max_features = 7.

#### 6.2.5 Stacked ensemble

The stacking ensemble technique begins with training fundamental classifiers using the provided dataset, followed by training an integrated classifier to incorporate the predictions of the other participants. The stacking approach works by training a meta-model (also known as the final estimator) on the predictions made by the base models. This meta-model learns to optimally combine the outputs of the base models or ensemble of classifiers, effectively exploiting their complementary strengths and weaknesses to produce a more accurate final prediction.

The Stacking Ensemble_1 approach can help overcome the limitations of individual classifiers. When a specific classifier fails to correctly classify instances from a particular region due to incomplete or inaccurate learning of the feature space, the second-level meta-classifier can learn from the behavior of the other base classifiers and use this information to correct the shortcomings of the individual models. This allows the stacking ensemble to leverage the strengths of the different classifiers and mitigate their individual weaknesses, leading to improved overall classification performance. The effectiveness of stacking is demonstrated by its capacity to generate superior outcomes compared to any individual classifier used. As a result, stacking has been employed to enhance the predictions of supervised learning tasks for both classification and regression.

In this study, we have designed two Stacked Ensemble models. The first, Stacked Ensemble_1, is created by ensembling the AdaBoost Classifier and XGBoost Classifier, with the final estimator being the RFC. These base models are expected to have different strengths and weaknesses, which are desirable for Stacked Ensemble_1. The Random Forest Classifier acts as the final estimator, allowing it to learn how to optimally leverage the predictions of the base models. The AdaBoost and XGBoost models may excel at different types of patterns, while the Random Forest serves as the final arbiter, learning to weigh the outputs of the base models effectively.

The second model, Stacked Ensemble_2, is constructed by combining a variety of base learners: Logistic Regression, XGBoost, Light Gradient Boosting, and Extra Trees Classifier. The idea and novelty behind Stacked Ensemble_2 are to build models with different decision boundaries (linear and non-linear) to increase overall diversity and improve performance.

The advantage of Stacked Ensemble_2 is that it uses a complex model like XGBoost, LGBM, or Extra Tree while adding a simpler model like LR, making the final decision process more interpretable for the ensemble model. In this case, Logistic Regression is added as a basic interpretable classifier, serving as the baseline. RFC is used as a final estimator to combine the predictions of both simple and complex models, improving robustness and reducing the likelihood of overfitting. The stacking ensemble approach often outperforms individual base models, as it effectively harnesses the complementary strengths of the different algorithms to achieve superior predictive performance (Table 13).

**Table 13:** Proposed models architecture.

Modeling building steps	Stacked Ensemble_1	Stacked Ensemble_2
Data preprocessing	Data cleaning IPv4 address conversion Encoding URL One-hot encoding Label encoding	Data cleaning IPv4 address conversion Encoding URL One-hot encoding Label encoding
Feature engineering	Shuffling data Variance threshold SMOTE (Balances class distributions)	Shuffling data Variance threshold SMOTE (Balances class distributions)
Base models	AdaBoost classifier XGBoost classifier	XGBoost LGBM Extra trees classifier Logistic regression
Final estimator	Random forest	Random forest

## 7 Results and analysis

### 7.1 Model performance

In this paper, we employed different models and algorithms for IoT cyberattack detection and classification systems to understand and predict misuse behavior. There are two common approaches used: machine learning and ensemble learning models. Both focus on applying classification techniques, with the former aiming to classify network traffic into different categories and the latter predicting the type of incident that occurred.

On the other hand, ensemble learning models utilize a combination of multiple machine learning models to learn complex patterns and relationships within Oil and Gas ICS and IoT data. In this comparison, we examined the performance of ensemble learning models in the context of Oil and Gas ICS and IoT cyberattack detection and classification systems by evaluating their respective accuracies and predictive capabilities. This analysis aimed to gain insights into the strengths and limitations of each approach and identify the models that provide the most promising results. The models examined include Bagging, AdaBoost, Gradient Boosting, XGBoost, Extra Tree, Random Forest, and Stacked Ensemble. The Edge-IIOT dataset for this work is split into 80% for training and 20% for testing. The hardware used for training and testing the different machine learning models on the dataset includes Google Cloud GPU.

In this work, the hyperparameters of models were tuned using Grid Search. Grid Search is used to perform an exhaustive search over a specified parameter grid to find the best combination of hyperparameters for the models. It works by systematically evaluating every possible combination of the hyperparameters defined in the param_grid dictionary, using cross-validation to ensure the robustness of the tuning process. This allows us to optimize the model’s performance by identifying the best combination of hyperparameters for our problem. The number of cross-validation folds used during the search was set to cv. = 5. The cross-validation approach employed by GridSearchCV splits the data into multiple folds, trains the model on each fold, and evaluates its performance using a specified scoring metric. This enables the algorithm to identify the set of hyperparameters that yields the best average score across the cross-validation folds, which are considered the “best” hyperparameters for the given model (Verma and Yadav, 2024).

Once the grid search is complete, the code retrieves the best hyperparameters and the corresponding best score from the GridSearchCV object. The main advantages of using GridSearchCV are its comprehensiveness and systematic approach to hyperparameter tuning, the robustness provided by cross-validation, and the abstraction of the manual process of trying different hyperparameter combinations, making the code more modular and easier to maintain (Nashaat, 2023). Based on the model trained and tested with hyperparameters tuned using Grid Search, we will now explain the results of all models in detail.

#### 7.1.1 Stacked ensemble models

##### 7.1.1.1 Stacked ensemble 1

A standard Stacked_Ensemble_1 model has been developed, where Adaboost and Xgboost are used as base models, with Random Forest as the final estimator. The Stacked_Ensemble_1 approach often outperforms individual base models, with overall training and testing accuracy at 96%. There have been fewer misclassifications, indicated by high Precision, Recall, and F1-Score. The model achieves a high true positive rate (TPR) of 94%, demonstrating strong sensitivity, and a low false positive rate (FPR) of 27%, with very few false alarms. The AUROC is 100%, demonstrating excellent discrimination capability between classes.

This has been compared with boosting models: AdaBoost, XGBoost, and RFC. The AdaBoost classifier shows moderate performance with an overall accuracy of approximately 54%, indicating limited effectiveness in correct classifications. High rates of False Positives and False Negatives, as reflected in the classification report’s Precision, Recall, and F1-Score, suggest issues with model reliability. The true positive rate (TPR) is 40%, meaning the model correctly identifies 40% of actual positives. The false positive rate (FPR) is 3%, which is relatively low but still contributes to some misclassification. The AUROC is 93%, indicating good discrimination ability, though overall accuracy remains modest.

The XGBoost model demonstrates strong performance with the following metrics: a training accuracy of 95% and a testing accuracy of 96%. The model performed with low false positives and false negatives, as shown by high Precision, Recall, and F1-Score. The true positive rate (TPR) is 94%, indicating effective identification of positive cases. The false positive rate (FPR) is 0.31%, reflecting very few false positives. The AUROC is 100%, showing excellent discriminative ability. Overall, XGBoost exhibits robust and reliable performance, balancing high accuracy with minimal misclassification.

The Random Forest Classifier (RFC) demonstrates excellent predictive performance, primarily driven by optimal hyperparameter tuning identified through Grid Search. The model achieved an accuracy of 96.00%, indicating high overall correctness. It minimized misclassifications, as reflected in elevated Precision, Recall, and F1-Score. The model achieved a true positive rate (TPR) of 96%, showing strong sensitivity in identifying positive cases. In addition, the false positive rate (FPR) is 21%, indicating very few false positives. Finally, an AUROC of 100% demonstrates outstanding discrimination capability. These results suggest that the optimized hyperparameters effectively control model complexity, leading to a robust and reliable classifier with minimal errors. The hyperparameters of the two models have been tuned using Grid Search to obtain the best parameters for model training and analysis toward an ensemble approach. The results have been tabulated as shown in Table 14.

**Table 14:** Classification model results–Stacked_Ensemble 1.

Models	Accuracy	Precision	Recall	F1 Score	TPR	FPR	AUROC
Stacked Ensemble_1	96%	95%	94%	94%	94%	27%	100%
AdaBoost	54%	54%	54%	43%	40%	3%	93%
XGBoost	96%	96%	96%	95%	94%	0.31%	100%
Random forest classifier	97%	95%	96%	95%	96%	21%	100%

The analysis of aggregated performance over repeated cross-validation reveals distinct profiles for each model. AdaBoost, while exceptionally fast with an average time of 24.06 (±0.78) seconds, consistently delivered very poor accuracy at 20.00% (±0.00), rendering its speed irrelevant. In contrast, XGBoost achieved high and stable accuracy at 95.28% (±0.10%) with a quick average execution time of 33.67 (±0.53) seconds. The Random Forest Classifier (RFC) emerged as the top performer in terms of accuracy, boasting the highest mean of 97.19% (±0.06%) with remarkable consistency; however, its average time of 52.36 (±38.78) seconds showed significant variability due to outlier runs. Lastly, the Stacked_Ensemble_1 yielded a high accuracy of 95.69% (±0.28%), but at a substantial computational cost, averaging 1034.71 (±527.70) seconds, making it an order of magnitude slower than the other models with considerable demand variability (Table 15).

**Table 15:** Mean ± SD over repeated runs and paired *t*-test for Stacked_Ensemble_1 models.

Models performance of repeated cross-validation Parameters: n_repeats = 3, n_splits = 5, scoring = ‘accuracy’		
Model name	Accuracy	Time (Sec)
Run 1/3		
AdaBoost	20.00%	25.03
XGBoost	95.30%	33.26
RFC	97.20%	24.94
Stacked_Ensemble_1	95.95%	300.16
Run 2/3		
AdaBoost	20.00%	24.05
XGBoost	95.27%	33.34
RFC	97.19%	107.20
Stacked_Ensemble_1	95.61%	1516.13
Run 3/3		
AdaBoost	20.00%	23.11
XGBoost	95.26%	34.42
RFC	97.18%	24.94
Stacked_Ensemble_1	95.53%	1287.85
Summary over repeated CV		
AdaBoost	20.00% ± 0.00	24.06 ± 0.78
XGBoost	95.28% ± 0.10%	33.67 ± 0.53
RFC	97.19% ± 0.06%	52.36 ± 38.78
Stacked_Ensemble_1	95.69% ± 0.28%	1034.71 ± 527.70

Paired statistical *t*-tests Stacked_Ensemble_1 vs. Baselines			
Model name	*t*_Stat	*p*_value	*p*_value < 0.05
AdaBoost	1005.1017	0.000000	Significant
XGBoost	6.0744	0.000029	Significant
RFC	−21.0935	0.000000	Significant

Paired statistical T-tests further elucidated these differences: the Stacked_Ensemble_1 was overwhelmingly and significantly superior to AdaBoost (P_value = 0.000000) and demonstrated a statistically significant, albeit small, improvement over XGBoost (P_value = 0.000029) in accuracy. However, a critical finding was that the Random Forest Classifier statistically significantly outperformed the Stacked_Ensemble_1 (P_value = 0.000000), indicating that despite its complexity and high computational cost, the ensemble could not achieve the accuracy level of the best single model (Tables 16, 17).

**Table 16:** Classification report for all the Stacked_Ensemble_1 models.

Stacked Ensemble_1 (Test data)	Adaboost (Test data)
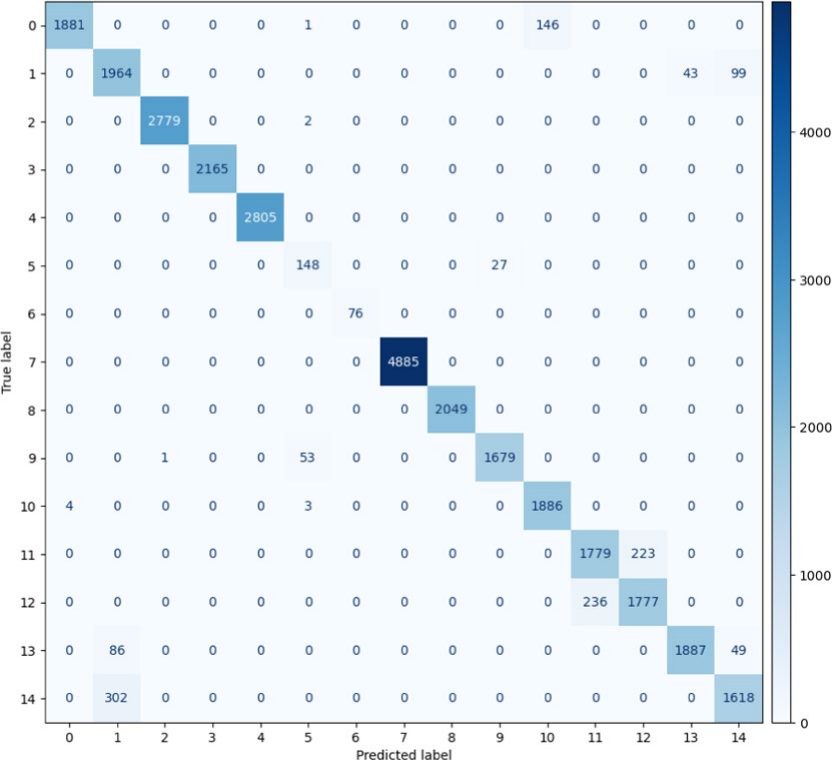	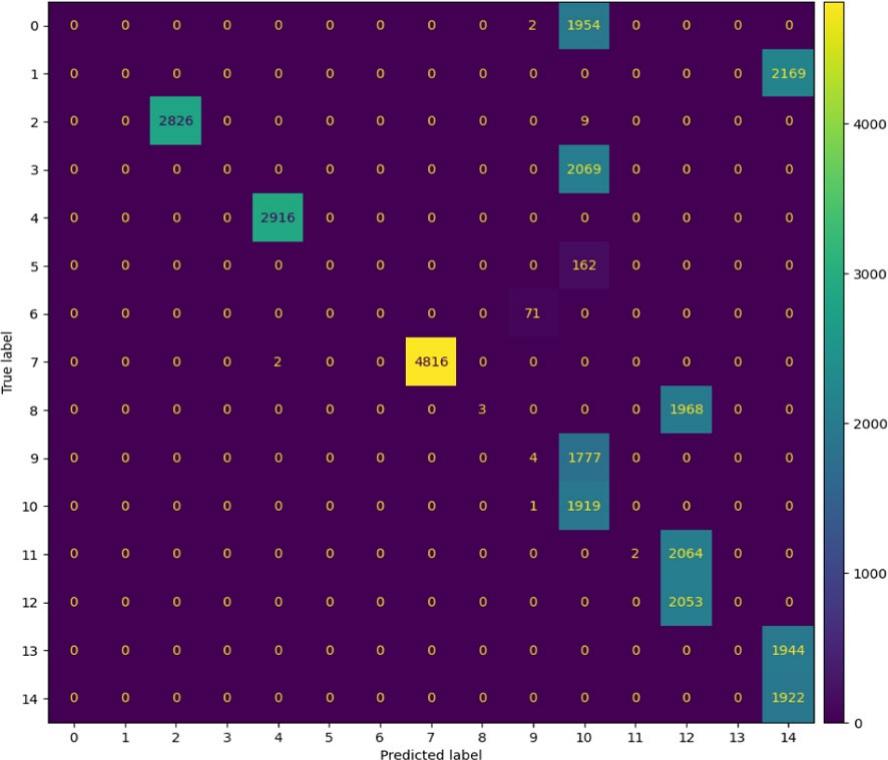

XGboost (Test data)	Random forest classifier (Test data)
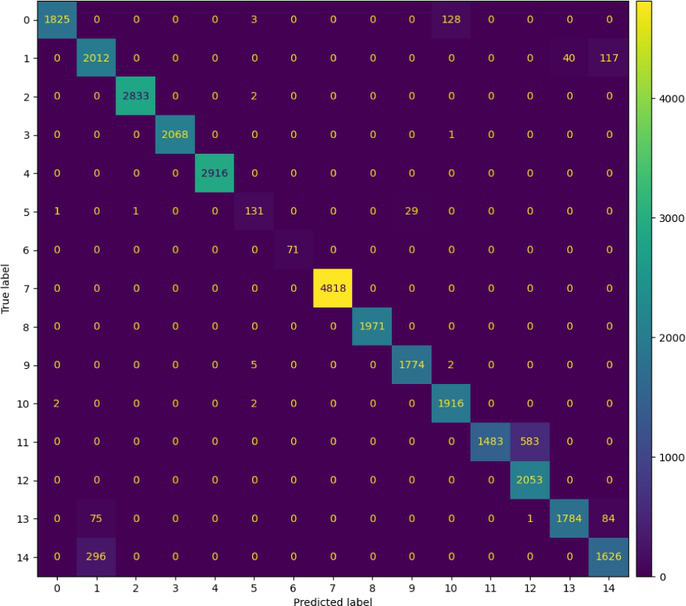	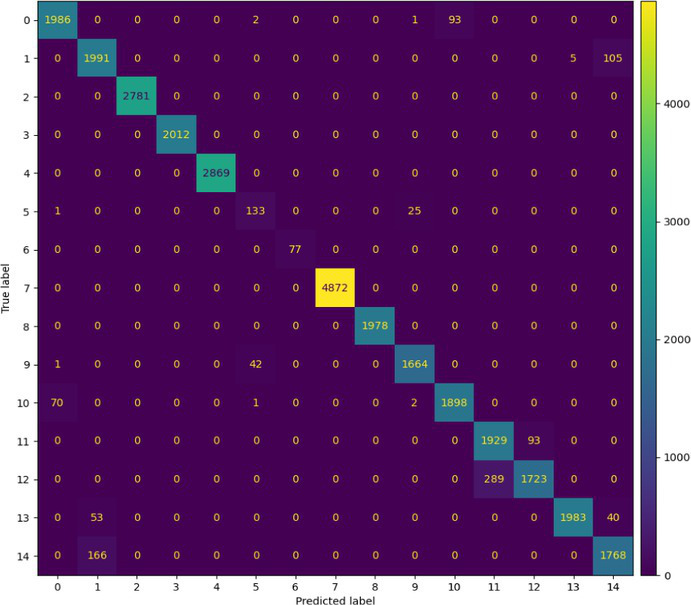

**Table 17:** Precision@k per-class performance results for Stacked_Ensemble_1.

Precision@k per-class performance												
Class name	Stacked Ensemble_1 (%)			Adaboost (%)			XGboost (%)			Random Forest classifier (%)		
	K = 3	K = 5	K = 10	K = 3	K = 5	K = 10	K3	K = 5	K = 10	K = 3	K = 5	K = 10
Backdoor	99.95%	99.95%	100%	0.15%	100%	100%	99.84%	100%	100%	99.84%	99.89%	100%
DDoS_HTTP	99.52%	100%	100%	100%	100%	100%	100%	100%	100%	100%	100%	100%
DDoS_ICMP	99.96%	100%	100%	99.56%	99.96%	99.96%	99.96%	99.96%	99.9%	99.96%	100%	100%
DDoS_TCP	100%	100%	100%	100%	100%	100%	100%	100%	100%	100%	100%	100%
DDoS_UDP	100%	100%	100%	100%	100%	100%	99.96%	99.96%	100%	100%	100%	100%
Fingerprinting	100%	100%	100%	99.39%	100%	100%	100%	100%	100%	99.39%	100%	100%
MITM	100%	100%	100%	100%	100%	100%	100%	100%	100%	100%	100%	100%
Normal	100%	100%	100%	100%	100%	100%	100%	100%	100%	100%	100%	100%
Password	100%	100%	100%	99.95%	100%	100%	100%	100%	100%	100%	100%	100%
Port_Scanning	99.59%	99.76%	99.88%	0.11%	100%	100%	99.94%	100%	100%	99.94%	99.94%	100%
Ransomware	99.78%	99.84%	99.94%	100%	100%	100%	99.95%	100%	100%	99.90%	99.90%	100%
SQL_injection	100%	100%	100%	100%	100%	100%	100%	100%	100%	100%	100%	100%
Uploading	100%	100%	100%	100%	100%	100%	100%	100%	100%	100%	100%	100%
Vulnerability_scanner	98.12%	100%	100%	100%	100%	100%	100%	100%	100%	100%	100%	100%
XSS	99.94%	100%	100%	100%	100%	100%	100%	100%	100%	100%	100%	100%

##### 7.1.1.2 Stacked Ensemble_2

In terms of Stacked_Ensemble_2, Logistic Regression (a linear model), XGBoost (a boosting model), LightGBM (a gradient boosting model), and Extra Trees (an ensemble of decision trees) were used as base models, with the Random Forest Classifier as the final estimator. The stacking ensemble approach often outperforms individual base models, as it can effectively harness the complementary strengths of the different algorithms to achieve superior predictive performance. The overall training and testing accuracy were 99 and 97%, respectively. This indicates a good level of performance in correctly identifying and classifying the different classes. There have been fewer misclassifications, as demonstrated by high Precision, Recall, and F1-Score. The model exhibits excellent performance with a high TPR of 97%, showing strong sensitivity, and a low FPR of 24%, indicating high specificity. The AUROC reported is 100%, showcasing outstanding discriminating ability.

This has been compared with individual classifiers: Logistic Regression (linear model), Boosting (XGBoost, LGB), Extra Trees Classifier, and RFC. The Logistic Regression model achieved an overall classification accuracy of 83%, correctly classifying 83% of instances. The model performs well across all classes but tends to classify the more frequent classes more accurately. Fewer misclassifications are reflected in high precision, recall, and F1 scores. A TPR of 81% indicates the model is highly effective at correctly identifying positive instances. Area under the receiver operating characteristic curve (AUROC) is 100%, signifying excellent discrimination ability between classes. The false positive rate is 1%, demonstrating the model’s high specificity with minimal false alarms.

The LightGBM (LGBM) classifier achieved a high overall prediction accuracy of 97%, demonstrating strong performance across most classes. The training accuracy was 98%, while the testing accuracy was 96%, indicating reliable generalization with minimal overfitting. The model shows a true positive rate (TPR) of 96%, reflecting excellent sensitivity, and a false positive rate (FPR) of 21%, which is very low, demonstrating high specificity. The AUROC is 100%, indicating exceptional discrimination between classes.

The Extra Trees classifier, after hyperparameter tuning via Grid Search, achieved impressive results with evaluations on both training and testing datasets. The training accuracy was exceptionally high at 100%, indicating an excellent fit to the training data, while the testing accuracy was 96%, demonstrating strong generalization to unseen data. The model showed a true positive rate (TPR) of 95%, indicating high sensitivity in detecting attacks, and a false positive rate (FPR) of 27%, reflecting good specificity. Finally, the AUROC is 99%, showing outstanding model discriminative ability. The results of stacked ensemble_2, along with other algorithms used, are tabulated, which include Logistic Regression (linear model), XGBoost (boosting model), LightGBM (gradient boosting model), Extra Trees (ensemble of decision trees), and Random Forest Classifier (Tables 18, 19).

**Table 18:** Classification model results for Stacked_Ensemble_2.

Models	Accuracy	Precision	Recall	F1 Score	TPR	FPR	AUROC
Stacked Ensemble_2	97%	97%	97%	97%	97%	24%	100%
Logistic regression	83%	84%	83%	83%	81%	1%	100%
XGboost	96%	96%	96%	95%	94%	0.31%	100%
LGBM	96%	95%	96%	95%	96%	21%	100%
Extra tree classifier	96%	93%	95%	94%	95%	27%	99%
Random forest classifier	96%	95%	96%	95%	96%	21%	100%

**Table 19:** Mean ± SD over repeated run and paired *t*-test for Stacked_Ensembled_2 models.

Models performance of repeated cross-validation Parameters: n_repeats = 3, n_splits = 5, scoring = ‘accuracy’		
Model name	Accuracy	Time (Sec)
Run 1/3		
Logistic regression	35.19%	109.98
XGBoost	95.30%	36.99
LGBM	97.26%	45.21
Extra tree classifier	97.00%	42.41
RFC	97.20%	32.13
Stacked_Ensemble_2	97.38%	1247.70
Run 2/3		
Logistic regression	34.95%	111.78
XGBoost	95.27%	37.14
LGBM	97.26%	40.51
Extra tree classifier	96.99%	40.17
RFC	97.19%	30.97
Stacked_Ensemble_2	97.36%	1227.50
Run 3/3		
Logistic regression	34.61%	112.04
XGBoost	95.26%	37.60
LGBM	97.25%	43.15
Extra tree classifier	96.97%	39.54
RFC	97.18%	31.37
Stacked_Ensemble_2	97.37%	1227.67
Summary over repeated CV		
Logistic regression	34.91% ± 0.49%	111.27 ± 0.92
XGBoost	95.28% ± 0.10%	37.24 ± 0.26
LGBM	97.26% ± 0.07%	42.96 ± 1.92
Extra tree classifier	96.99% ± 0.07%	40.70 ± 1.23
RFC	97.19% ± 0.06%	31.49 ± 0.48
Stacked_Ensemble_2	97.37% ± 0.07%	1234.29 ± 9.48

Paired statistical *t*-tests Stacked_Ensemble_1 vs. baselines			
Model name	*t*_Stat	*p*_value	*p*_value < 0.05
Logistic regression	469.2706	0.000000	Significant
XGBoost	85.0308	0.000000	Significant
LGBM	10.7371	0.000000	Significant
Extra tree classifier	22.3671	0.000000	Significant
RFC	13.6724	0.000000	Significant

The aggregated performance data reveals that while Logistic Regression delivered poor accuracy at 34.91% (±0.49%) despite a relatively slow average time of 111.27 (±0.92) seconds, XGBoost, LGBM, Extra Tree Classifier, and RFC all achieved high and consistent accuracies ranging from 95.28 to 97.26% within efficient timeframes, with RFC being the most time-efficient at 31.49 (±0.48) seconds. Crucially, the Stacked_Ensemble_2 recorded the highest mean accuracy of 97.37% (±0.07%) with excellent consistency; however, this superior performance came at a significant computational cost, averaging an extremely slow 1234.29 (±9.48) seconds. Paired statistical T-tests confirmed that the Stacked_Ensemble_2 statistically and significantly outperformed every baseline model in accuracy, including the highly performing RFC and LGBM, thereby establishing its predictive superiority despite its substantial time investment (Tables 20, 21).

**Table 20:** Classification report for all the Stacked_Ensemble_2 models.

Stacked Ensemble_2 (Test data)	Logistic regression (Test data)
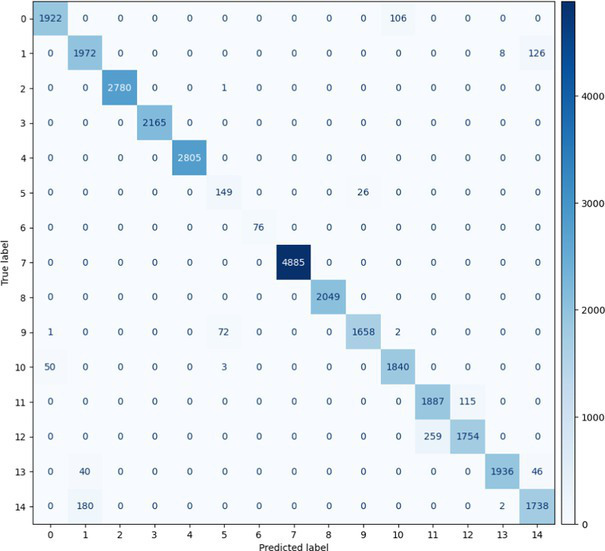	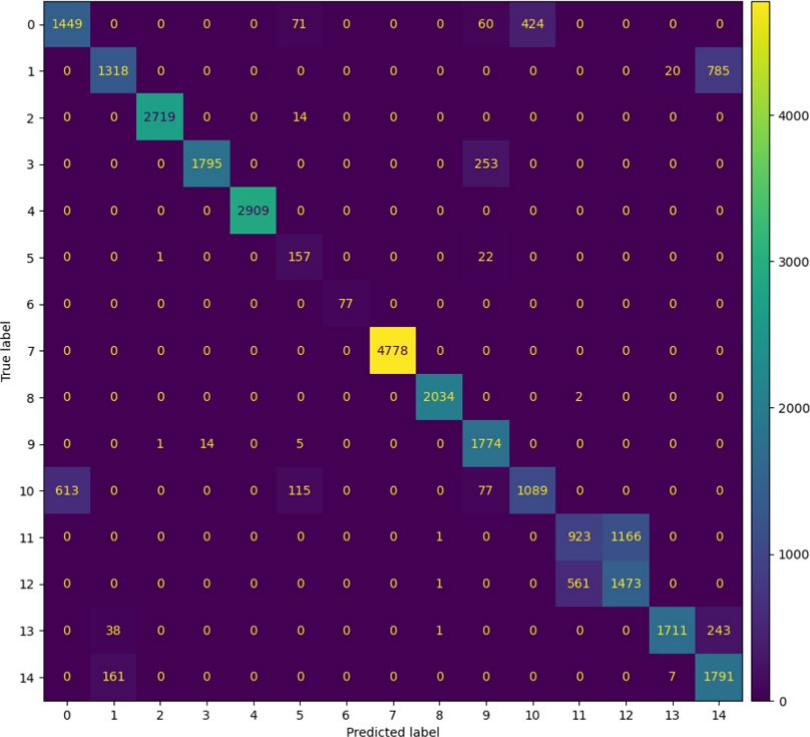

XGboost (Test data)	LGBM (Test data)
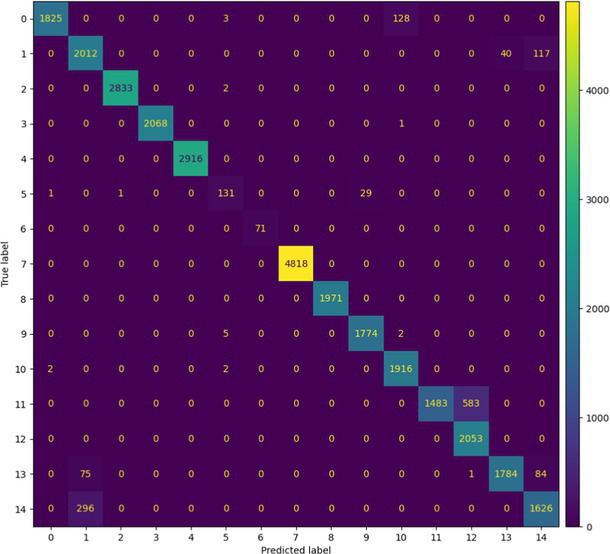	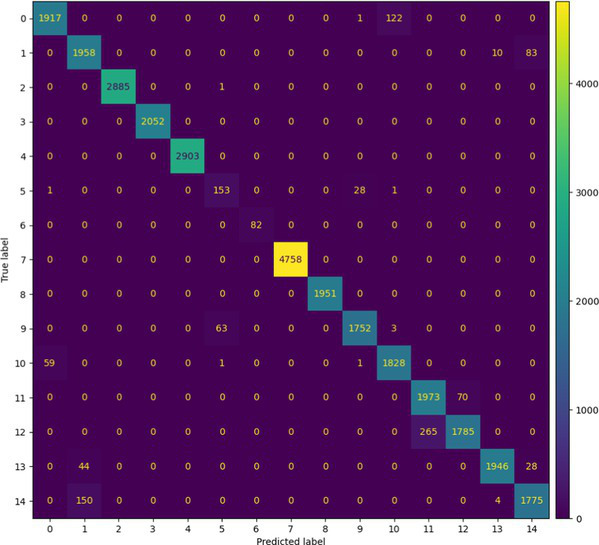

Extra tree classifier (Test data)	Random forest classifier (Test data)
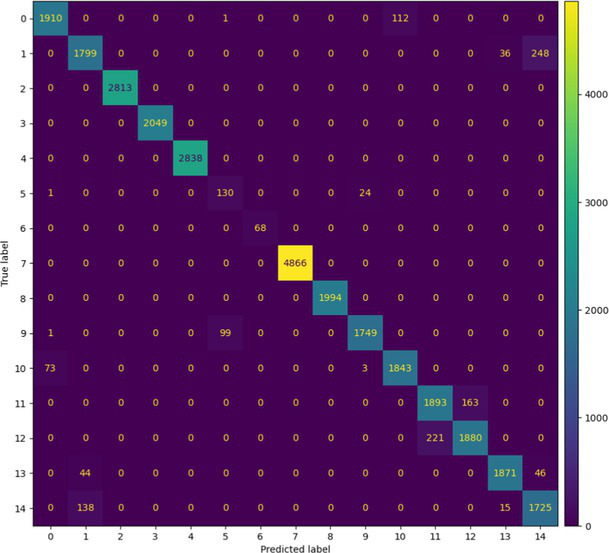	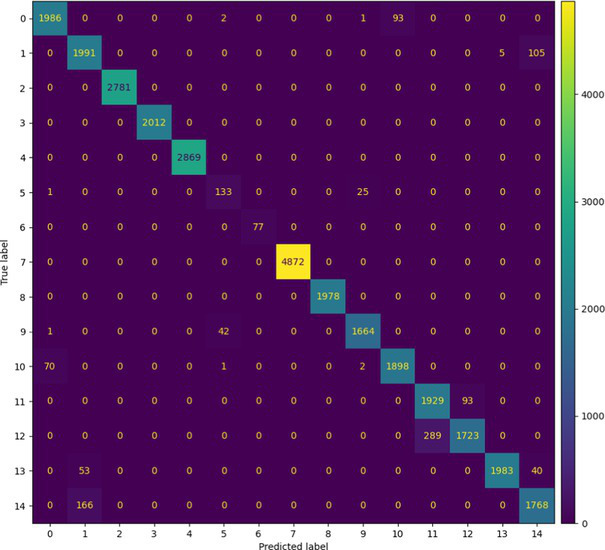

**Table 21:** Precision@k per-class performance results for Stacked_Ensemble_2.

Precision@k per-class performance												
Class name	Stacked Ensemble_2 (%)			Logistic regression (%)			LGBM (%)			Extra tree classifier (%)		
	K = 3	K = 5	K = 10	K = 3	K = 5	K = 10	K3	K = 5	K = 10	K = 3	K = 5	K = 10
Backdoor	100%	100%	100%	93.97%	100%	100%	99.84%	100%	100%	98.76%	98.81%	98.81%
DDoS_HTTP	100%	100%	100%	100%	100%	100%	100%	100%	100%	96.66%	96.66%	96.66%
DDoS_ICMP	100%	100%	100%	100%	100%	100%	100%	100%	100%	100%	100%	100%
DDoS_TCP	100%	100%	100%	96.43%	100%	100%	100%	100%	100%	100%	100%	100%
DDoS_UDP	100%	100%	100%	100%	100%	100%	100%	100%	100%	100%	100%	100%
Fingerprinting	98.85%	98.85%	100%	100%	100%	100%	100%	100%	100%	96.10%	96.10%	98.70%
MITM	100%	100%	100%	100%	100%	100%	100%	100%	100%	100%	100%	100%
Normal	100%	100%	100%	100%	100%	100%	100%	100%	100%	100%	100%	100%
Password	100%	100%	100%	100%	100%	100%	100%	100%	100%	100%	100%	100%
Port_Scanning	99.71%	100%	100%	99.94%	99.94%	100%	99.94%	100%	100%	99.94%	99.94%	100%
Ransomware	99.89%	100%	100%	94.93%	100%	100%	99.94%	100%	100%	98.72%	98.72%	100%
SQL_injection	100%	100%	100%	100%	100%	100%	100%	100%	100%	98.80%	100%	100%
Uploading	100%	100%	100%	100%	100%	100%	100%	100%	100%	97.22%	100%	100%
Vulnerability_scanner	100%	100%	100%	100%	100%	100%	100%	100%	100%	100%	100%	100%
XSS	100%	100%	100%	100%	100%	100%	100%	100%	100%	100%	100%	100%

##### 7.1.1.3 Bagging

The described bagging ensemble, comprising 500 decision trees with a maximum depth of 12 and trained on bootstrap samples of 300 instances, exhibits excellent performance in attack classification. The high training accuracy (93%) and test accuracy (94%) indicate robust generalization. The high precision, recall, and F1-score suggest that the model effectively balances false positives and false negatives, minimizing misclassification. The model exhibits a true positive rate (TPR) of 93% and a false positive rate (FPR) of 0.42%. The AUROC is 100%, indicating near-perfect discrimination capability between classes. Overall, this setup demonstrates a well-tuned ensemble model capable of reliable detection in the given classification task, with strong potential for deployment in real-world scenarios where high accuracy and low false positive rates are critical. The bagging model has been compared with the decision tree model.

In terms of the decision tree, where grid search is employed for hyperparameter tuning, the search results do not directly mention how ‘gini’ and ‘entropy’ are used. However, the general principle is that the decision tree algorithm selects the split attribute that maximizes information gain (for entropy) or minimizes the Gini index at each node of the tree. This process is carried out in a greedy, top-down manner, with the best split chosen at each step without considering the long-term impact on the entire tree (Banerjee, 2023; Dash, 2023). The decision tree model demonstrates strong performance in classifying attacks, achieving an overall training accuracy of 98% and a testing accuracy of 96%. The high accuracy, along with metrics such as precision, recall, and F1-score, indicates a low rate of misclassification. Specifically, the True Positive Rate (recall) is 93%, and the False Positive Rate is very low at 0.26%. The AUROC of 98% suggests the model has excellent discriminative ability between attack and non-attack instances, making it a reliable choice for attack classification tasks. The results of both the bagging ensemble model and the decision tree are tabulated in Tables 22, 23.

**Table 22:** Classification model results – bagging.

Models	Accuracy	Precision	Recall	F1 Score	TPR	FPR	AUROC
Bagging	94%	91%	93%	91%	93%	0.42%	100%
Decision tree	96%	95%	96%	95%	96%	0.26%	98%

**Table 23:** Precision@k per-class performance results for bagging.

Precision@k per-class performance						
Class name	Bagging (%)			Decision tree (%)		
	K = 3	K = 5	K = 10	K = 3	K = 5	K = 10
Backdoor	99.22%	100%	100%	99.17%	99.17%	99.17%
DDoS_HTTP	100%	100%	100%	98.57%	98.57%	98.57%
DDoS_ICMP	99.89%	99.96%	100%	99.89%	99.89%	99.89%
DDoS_TCP	100%	100%	100%	100%	100%	100%
DDoS_UDP	100%	100%	100%	100%	100%	100%
Fingerprinting	100%	100%	100%	90.90%	91.51%	99.39%
MITM	100%	100%	100%	100%	100%	100%
Normal	100%	100%	100%	100%	100%	100%
Password	100%	100%	100%	100%	100%	100%
Port_Scanning	99.88%	99.88%	100%	98.45%	98.45%	100%
Ransomware	99.95%	100%	100%	99.100%	99.10%	100%
SQL_injection	100%	100%	100%	99.8%	100%	100%
Uploading	100%	100%	100%	99.50%	100%	100%
Vulnerability_scanner	100%	100%	100%	100%	100%	100%
XSS	100%	100%	100%	100%	100%	100%

### 7.2 Computation of evaluation metrics (FPR, TPR, and AUROC)

To evaluate the classification performance across all attack categories, the true positive rate (TPR), false positive rate (FPR), and area under the receiver operating characteristic curve (AUROC) were computed for each class individually and summarized using macro-averaging to provide a balanced view of performance across both majority and minority classes.

The TPR, equivalent to recall, was computed for each class as the ratio of correctly identified positive instances (true positives) to the total actual positives (true positives plus false negatives). Similarly, the FPR was derived using per-class precision, recall, and support values, representing the proportion of negative instances incorrectly classified as positive. For each class, true positives, false positives, false negatives, and true negatives were estimated using precision and recall relationships. The macro-averaged FPR and TPR were then obtained by taking the mean values across all 15 classes. The models’ performance was calculated as follows:


$$\mathrm{FPR}=\frac{\mathrm{FP}}{\left(\mathrm{FP}+\mathrm{TN}\right)},\mathrm{TPR}=\frac{\mathrm{FP}}{\left(\mathrm{TP}+\mathrm{FN}\right)}$$

For the AUROC, class-wise probabilities were obtained using the model’s predict_proba() function, and the One-vs-Rest (OvR) strategy was applied to compute the area under the ROC curve for each class. The final AUROC score was calculated using the roc_auc_score() function from scikit-learn with multi_class = ‘ovr’, which aggregates the individual class AUROC values into a single representative performance measure. This combination of metrics provides a comprehensive assessment of the model’s discriminative capability across all attack categories, accounting for both correctly and incorrectly classified instances in the multi-class setting.

Having analyzed the performance of all the machine learning models, which included individual classifiers like Logistic Regression and decision tree, as well as ensemble machine learning models such as Bagging, Boosting, Random Forest, Extra Trees, and two Stacked Ensemble models, we examined the performance of the ensemble classification models in the context of Oil and Gas ICS and IoT cyberattack detection and classification systems by evaluating their respective accuracies and predictive capabilities to gain insights into the strengths and limitations of each approach in identifying the models that provide the most promising results.

The comparative results presented in Table 24 clearly demonstrate the superior performance of the stacked ensemble_2 model over stacked ensemble_1, ensemble and the individual machine learning algorithms for the ICS & IoT attack in oil and gas with an overall accuracy of 97%. The next best performing models have been stacked ensemble_1, Extra Tree, LGBM, Logistic Regression and Random Forest with an accuracy of 96, 96, 96, 97 and 96, respectively. Even though the performance of stacked Ensemble_2 was the best, there is a need to analyze the computational cost for deployment in real time in Edge-IIoT. All the runtime experiments were conducted on a MacBook Pro (Apple M1, 8 GB RAM) using an Anaconda-managed environment; it was measured using wall-clock time through Python’s built-in time module. Specifically, the total training duration was calculated by recording the start and end times of the execution process using the command (start_time = time.time() and training_time = time.time() - start_time) before model training and computing the elapsed time after completion. The reported runtime reflects the total training time rather than per-fold or per-run averages then the result was converted to minutes (Table 25).

**Table 24:** Performance of the models in detecting ICS and IoT attacks.

Individual ML models		Ensemble models	
Algorithm	Accuracy	Algorithm	Accuracy
DT	96%	Bagging	94%
		AdaBoost	54%
Logistic regression	83%	XGBoost	96%
		LGBM	96%
RFC	96%	Stacked Ensemble_1	96%
Extra tree classifier	96%	Stacked Ensemble_2	97%

**Table 25:** Classification report for Stacked Ensemble_1 and Stacked Ensemble_1.

Classes no	Classification report–Stacked Ensemble_1				Classification report–Stacked Ensemble_2			
	Precision	Recall	F1-score	Support	Precision	Recall	F1-Score	Support
Backdoor	98%	94%	96%	1956	95%	96%	96%	1932
DDoS_HTTP	84%	94%	89%	2,169	90%	93%	92%	2025
DDoS_ICMP	100%	100%	100%	2,835	100%	100%	100%	2,832
DDoS_TCP	100%	100%	100%	2069	100%	100%	100%	2042
DDoS_UDP	100%	100%	100%	2,916	100%	100%	100%	3,005
Fingerprinting	91%	81%	86%	162	62%	86%	72%	160
MITM	100%	100%	100%	71	100%	100%	100%	76
Normal	100%	100%	100%	4,818	100%	100%	100%	4,840
Password	100%	100%	100%	1971	100%	100%	100%	1996
Port Scanning	98%	100%	99%	1781	99%	95%	97%	1755
Ransomware	94%	98%	96%	1920	96%	95%	95%	1916
SQL injection	89%	91%	90%	2066	87%	97%	91%	2079
Uploading	91%	89%	90%	2053	96%	85%	90%	2042
Vulnerability scanner	98%	94%	96%	1944	100%	96%	98%	2031
XSS	93%	84%	89%	1922	90%	91%	91%	1922
Model performance result after training								
Accuracy			96%	30,653			97%	30,653
Macro avg	96%	95%	95%	30,653	94%	96%	95%	30,653
Weighted Avg	96%	96%	96%	30,653	97%	97%	97%	30,653

**Figure 4:**
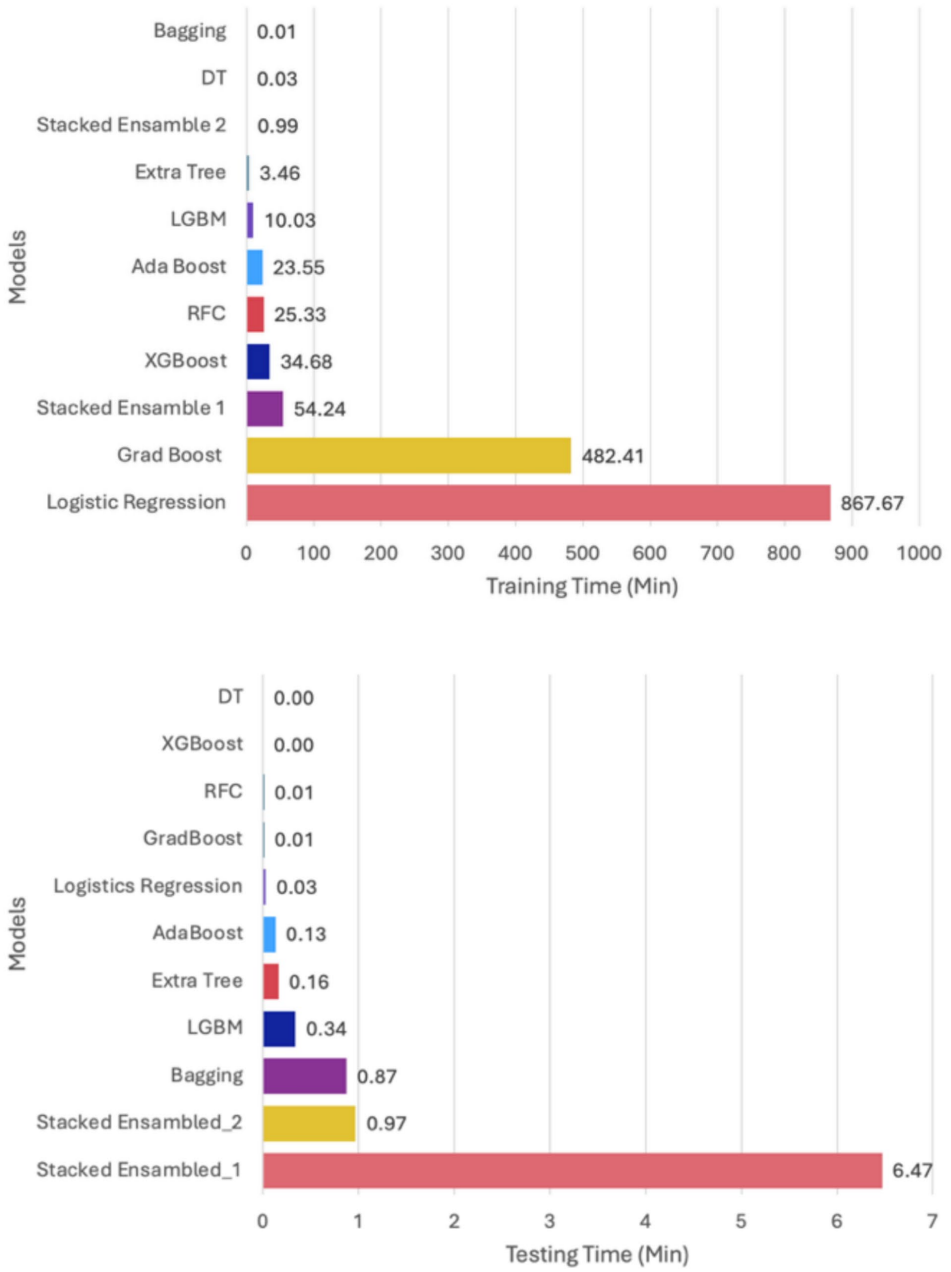
Training and testing time for each model.

The unusually long runtime observed for the Logistic Regression model (approximately 867 min) is primarily due to the computational intensity of exhaustive hyperparameter optimization combined with the large, multi-class dataset used in this study Figure 4. The model was trained using a GridSearchCV procedure, which systematically evaluates multiple combinations of hyperparameters—such as regularization strength (C), penalty type, and solver choice—across several cross-validation folds. This process requires fitting the model repeatedly, greatly increasing the total computation time. Additionally, the dataset was resampled using SMOTE to address class imbalance, further enlarging the training data and intensifying the optimization workload.

Classifying 15 attack categories required the use of the one-vs-rest (OvR) multi-class strategy, where a separate binary logistic model is trained for each class. This effectively multiplies the number of optimization problems being solved, as each sub-model iteratively computes gradient updates over all data points. Given that Logistic Regression relies on iterative numerical optimization, the presence of a high-dimensional feature space and a large sample size substantially increases convergence time. The trade-off on accuracy, train and testing time has been tabulated toward trade-off as shown in Table 26.

**Table 26:** Trade-off on the models between accuracy, training, and testing time in detecting ICS and IoT attacks.

Model	Accuracy (~%)	Training time	Testing time	Suitable for
Stacked Ensemble_2	97%	~54 min	~1 min	Highest accuracy: use if resources and time allow.
Stacked Ensemble_1	95%	~1 min	~6.5 min	Good balance of performance and efficiency.
Extra trees	~94.75%	~10 min	~0.16 min	Fast and robust; suitable for near real-time detection.
LightGBM	~94.9%	~3.46 min	~0.34 min	Very fast, efficient; suitable for real-time use.
XGboost	96%	34.68 min	0.00 min	Very fast inference with good balance of accuracy; suitable for applications requiring instant decision making.
AdaBoost	54%	23.55 min	0.13 min	Relatively poor performance in the current task. Suggests that inference can be performed quickly once the model is trained. Not reliable for critical applications like attack detection.
Random forest	~94–95%	~25 min	~0.01 min	Extremely fast inference; good for rapid deployment.
Logistic regression	~83%	~867 min (longest)	~0.03 min	Highly accurate but costly to train; best for offline.
Decision tree	~94–95%	~0.03 min	0.00 min	Very quick; suitable when speed is prioritized.
Bagging	~94–95%	~0.01 min	~0.87 min	Fast training/testing; suitable for quick deployment.

Models like stacked Ensemble_2 achieved top accuracy with increased training time and computational cost. For applications where training resources or time are limited, simpler models like Decision Trees or Bagging provide a good balance of accuracy (~94–95%) and much faster training. If rapid inference is critical (e.g., live attack detection), models like decision trees, LightGBM, or Random Forest are preferred. However, high inference speed often coincides with slightly lower accuracy compared to ensemble models like Ensemble_2. For quick deployment and iteration, simple models are advantageous; for maximum accuracy where training time is less critical, ensemble methods are better. Additionally, these stacked ensembles are preferred when training occurs on powerful, off-site servers, and inference speed and resource usage on the deployment device are less critical. Furthermore, the environment allows for longer training periods with acceptable inference latency. Otherwise, in severely resource-constrained IoT or edge environments, lighter models like stacked Ensemble_1 are more appropriate. In short, choosing ensemble models increases accuracy but at the cost of computational resources.

**Figure 5:**
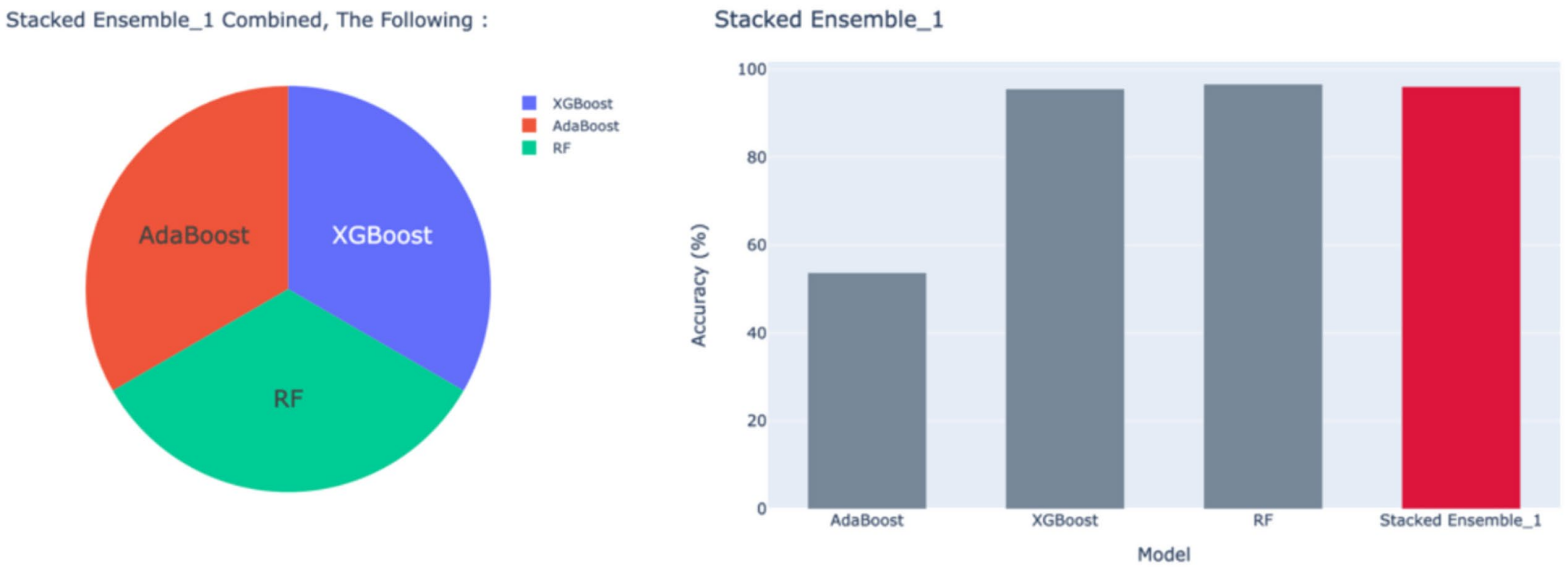
Comparison of Stacked Ensemble_1 results.

The stacked Ensemble_1 model, which includes Extra Tree Classifier, Random Forest, and Decision Tree, achieves an impressive 96% accuracy with marginal differences. The stacked ensemble method leverages the complementary strengths of multiple base learners, effectively combining their predictive power to produce superior results compared to any single model. The Stacking Ensemble showcases its ability to intelligently weigh the outputs of the base models (AdaBoost, XGBoost, and RF) to arrive at the final predictions, outperforming the individual component models, as shown in Figure 5.

**Figure 6:**
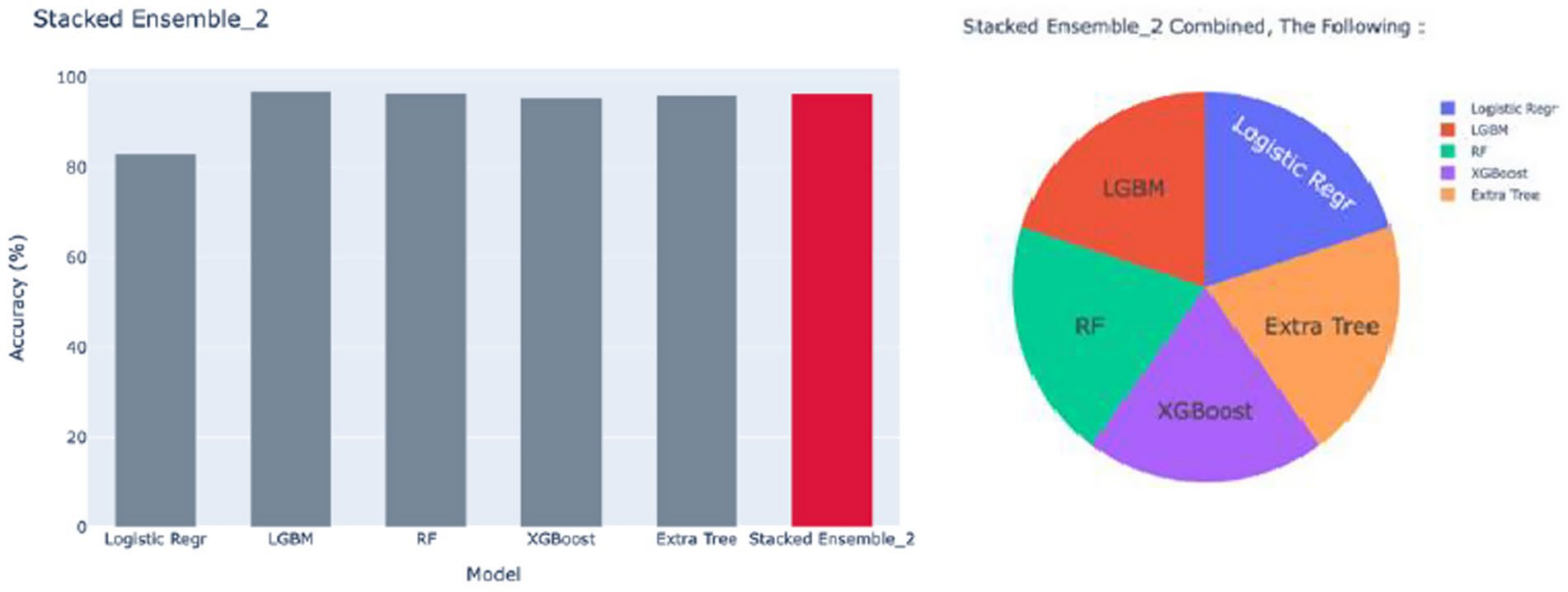
Comparison of Stacked_Ensemble_2 results.

The stacked Ensemble_2 model achieved an impressive 97% accuracy over other models, including Extra Trees, Random Forest, XGBoost, LGBM, and AdaBoost. The stacked ensemble method effectively combines the complementary strengths of multiple base learners to produce superior results compared to any single model. The Stacking Ensemble demonstrates its ability to intelligently weigh the outputs of both linear and non-linear base models to arrive at the final predictions, outperforming the individual component models, as shown Figure 6.

This suggests that the detection of oil and gas ICS and IoT systems involves complex underlying patterns and relationships that are better captured by ensemble methods, which can extract more meaningful information from the data by aggregating the diverse perspectives of multiple models to detect abnormal behavior before an incident occurs.

The clear superiority of the ensemble models, particularly Stacked Ensemble_2, makes them the more appropriate and reliable choices for oil and gas ICS and IoT cyberattacks. The ability of ensemble methods to harness the collective strengths of multiple algorithms sets them apart from individual machine learning models, making them the preferred solution for achieving high-performing and robust predictions in this context.

### 7.3 Component contribution analysis and models ablation study

To assess the contribution of each preprocessing and modeling component, an ablation study was conducted comparing baseline models to versions incorporating feature selection, data preprocessing, SMOTE balancing, and hyperparameter tuning. Results demonstrate that each step incrementally improved classification performance (Table 27).

**Table 27:** A details analysis about component contribution in detecting ICS and IoT attacks.

Model name	Model setup	Number of features	Accuracy	Precision	Recall	F1-score
DT	Base model: - No SMOTE - No Feature Engineering - No hyperparameter Tuning - No Grid Search	(152,196, 34)	0.89%	0.87%	0.87%	0.87%
	+ Data pre-processing + Feature Engineering + SMOTE	(153,264, 68)	0.96%	0.93%	0.95%	0.93%
	All + Hyperparameter Tuning (Our Experiment)	(153,264, 68)	96%	95%	96%	95%
RFC	Base model: - No SMOTE - No Feature Engineering - No hyperparameter Tuning - No Grid Search	(152,196, 34)	0.90%	0.88%	0.88%	0.88%
	+Data pre-processing + Feature Engineering + SMOTE	(153,264, 68)	0.96%	0.94%	0.95%	0.94%
	All + Hyperparameter Tuning (Our Experiment)	(153,264, 75)	96%	95%	96%	95%
Logistic Regression	Base model: - No SMOTE - No Feature Engineering - No hyperparameter Tuning - No Grid Search	(152,196, 34)	0.34%	0.23%	0.30%	0.25%
	+Data pre-processing + Feature Engineering + SMOTE	(153,264, 68)	0.32%	0.35%	0.33%	0.31%
	All + Hyperparameter Tuning (Our Experiment)	(153,264, 75)	83%	84%	83%	83%
Bagging	Base model: - No SMOTE - No Feature Engineering - No hyperparameter Tuning - No Grid Search	(152,196, 34)	0.90%	0.88%	0.88%	0.88%
	+Data pre-processing + Feature Engineering + SMOTE	(153,264, 68)	0.96%	0.93%	0.95%	0.94%
	All + Hyperparameter Tuning (Our Experiment)	(153,264, 68)	94%	91%	93%	91%
AdaBoost	Base model: - No SMOTE - No Feature Engineering - No hyperparameter Tuning - No Grid Search	(152,196, 34)	0.89%	0.87%	0.87%	0.87%
	+Data pre-processing + Feature Engineering + SMOTE	(153,264, 68)	0.96%	0.93%	0.95%	0.94%
	All + Hyperparameter Tuning (Our Experiment)	(153,264, 68)	54%	54%	54%	43%
XGboost	Base model: - No SMOTE - No Feature Engineering - No hyperparameter Tuning - No Grid Search	(152,196, 34)	0.91%	0.91%	0.89%	0.89%
	+Data pre-processing + Feature Engineering + SMOTE	(153,264, 68)	0.97%	0.95%	0.96%	0.95%
	All + Hyperparameter Tuning (Our Experiment)	(153,264, 68)	96%	96%	96%	95%
LGBM	Base model: - No SMOTE - No Feature Engineering - No hyperparameter Tuning - No Grid Search	(152,196, 34)	0.06%	0.27%	0.06%	0.06%
	+Data pre-processing + Feature Engineering + SMOTE	(153,264, 68)	0.97%	0.95%	0.96%	0.95%
	All + Hyperparameter Tuning (Our Experiment)	(153,264, 68)	96%	95%	96%	95%
Extra tree classifier	Base model: - No SMOTE - No Feature Engineering - No hyperparameter Tuning - No Grid Search	(152,196, 34)	0.88%	0.87%	0.87%	0.87%
	+ Data pre-processing + Feature Engineering + SMOTE	(153,264, 68)	0.96%	0.94%	0.95%	0.94%
	All + Hyperparameter Tuning (Our Experiment)	(153,264, 68)	96%	93%	95%	94%
Stacked Ensemble_1	Base model: - No SMOTE - No Feature Engineering - No hyperparameter Tuning - No Grid Search	(152,196, 34)	0.91%	0.92%	0.88%	0.89%
	+ Data pre-processing + Feature Engineering + SMOTE	(153,264, 68)	0.97%	0.94%	0.96%	0.95%
	All + Hyperparameter Tuning (Our Experiment)	(153,264, 68)	96%	95%	94%	94%
Stacked Ensemble_2	Base model: - No SMOTE - No Feature Engineering - No hyperparameter Tuning - No Grid Search	(152,196, 34)	0.91%	0.92%	0.88%	0.89%
	+ Data pre-processing + Feature Engineering + SMOTE	(153,264, 68)	0.97%	0.94%	0.96%	0.95%
	All + Hyperparameter Tuning (Our experiment)	(153,264, 68)	97%	97%	97%	97%

To assess the contribution of each base classifier within Stacked Ensemble_1 and Stacked Ensemble_2, we conducted an ablation study. Specifically, we re-trained the stacking model while systematically removing one base learner at a time. This allowed us to quantify the performance impact of each model on the ensemble’s overall accuracy and robustness (Table 28).

**Table 28:** Models ablation study on the detecting of ICS and IoT attacks.

Model name	Ablation scenarios	Accuracy	Precision	Recall	F1 score
Stacked Ensemble_1	*Stacked_Ensemble_1*	96%	96%	96%	96%
	No MetaLearner, Avg (AdaBoost + XGboost)	96%	96%	96%	95%
	Without AdaBoost	96%	96%	96%	96%
	Without_XGBoost	47%	38%	47%	40%
Stacked Ensemble_2	*Stacked_Ensemble_2*	97%	97%	97%	97%
	No MetaLearner, Avg (LR, XGB, LGBM, ExtraTrees)	96%	97%	96%	96%
	Without LogisticRegression	96%	97%	96%	97%
	Without XGBoost	97%	97%	97%	97%
	Without LGBM	96%	96%	96%	96%
	Without ExtraTrees	97%	97%	97%	97%

From the ablation study, it is clear that for Stacked Ensemble_1, Adaboost+Xgboost without the metalearner RFC and Xgboost with the metalearner RFC were responsible for driving the gain in Stacked Ensemble_1. The configuration without Xgboost followed by the metalearner resulted in a low gain. Thus, the Xgboost classifier has been the dominant classifier driving the gain. In terms of Stacked Ensemble_2, all classifiers contributed to the gain, regardless of the presence of the metalearner, which is RFC. Additionally, by removing certain classifiers while retaining the metalearner, the Stacked Ensemble_2 achieved a higher gain.

Additionally, model interpretability was enhanced by analyzing SHAP values, which revealed that flow-based and packet-related features had the highest contribution to distinguishing between types of attacks. This analysis provides insight into the model’s decision process and reinforces the validity of the selected features (Table 29).

**Table 29:** Shape analysis for all the individual models and proposed models to show important features for each model.

Model name	SHAP analysis for Top 20 feature (Test data)	Model name	SHAP analysis for top 20 feature (Test data)
DT	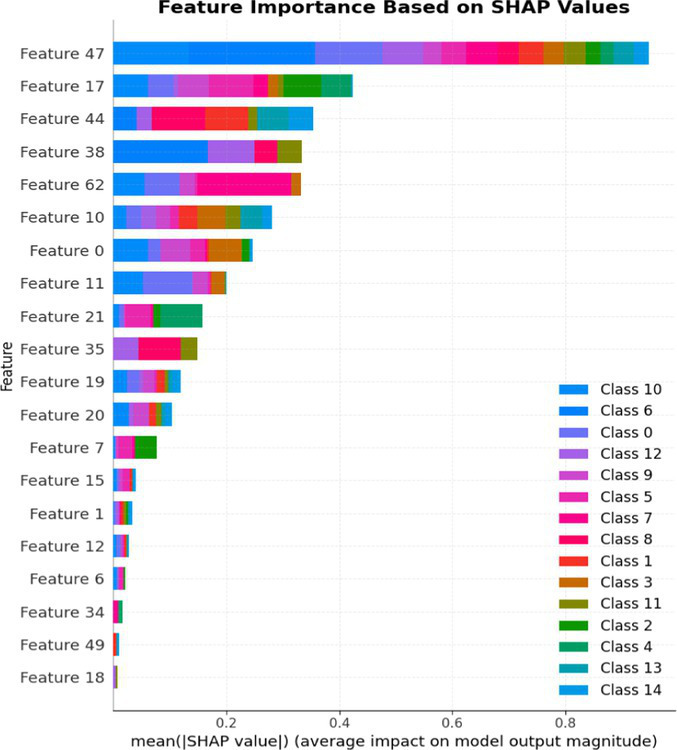	RFC	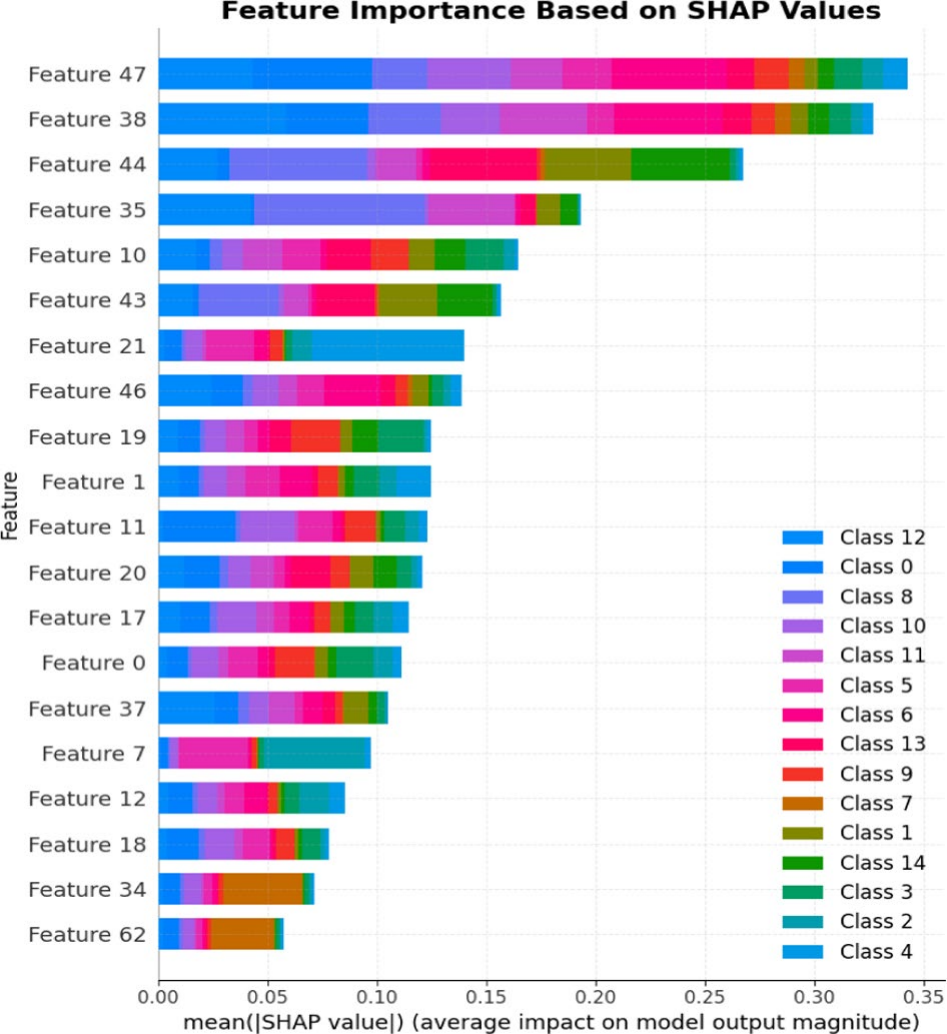
LR	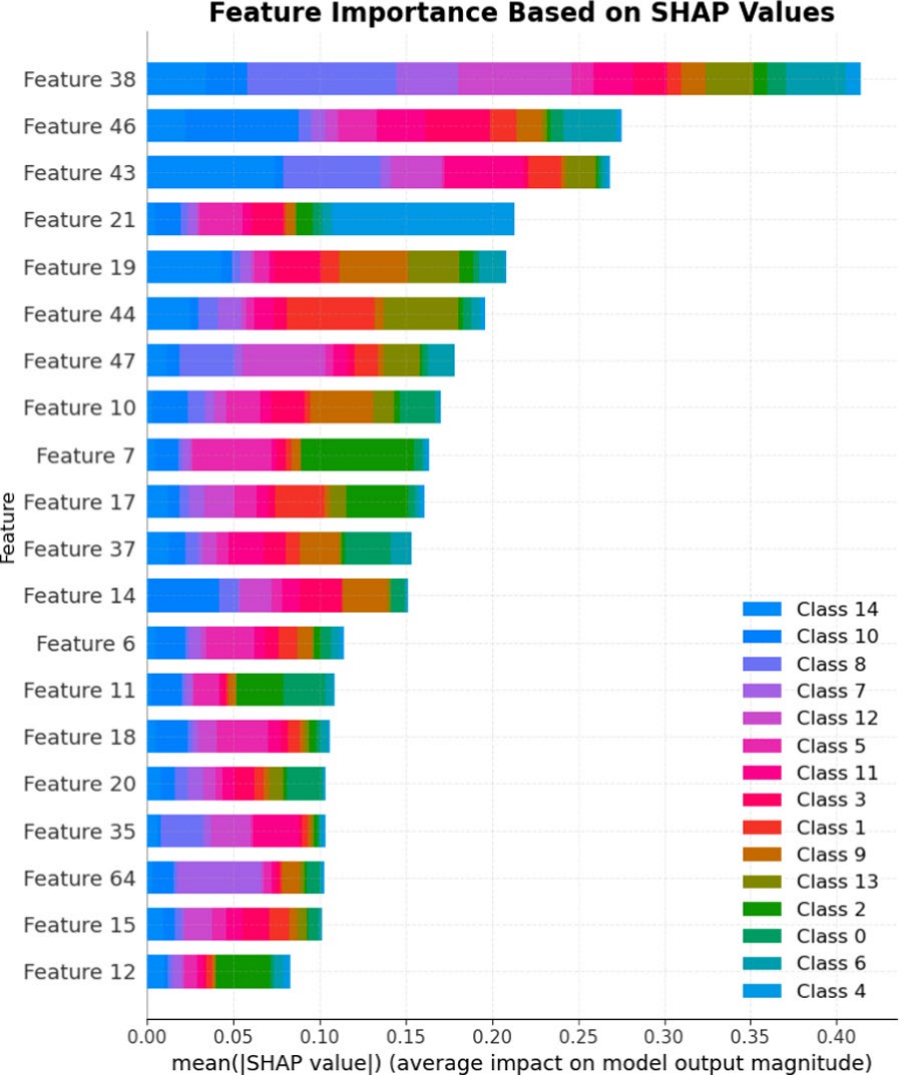	Bagging	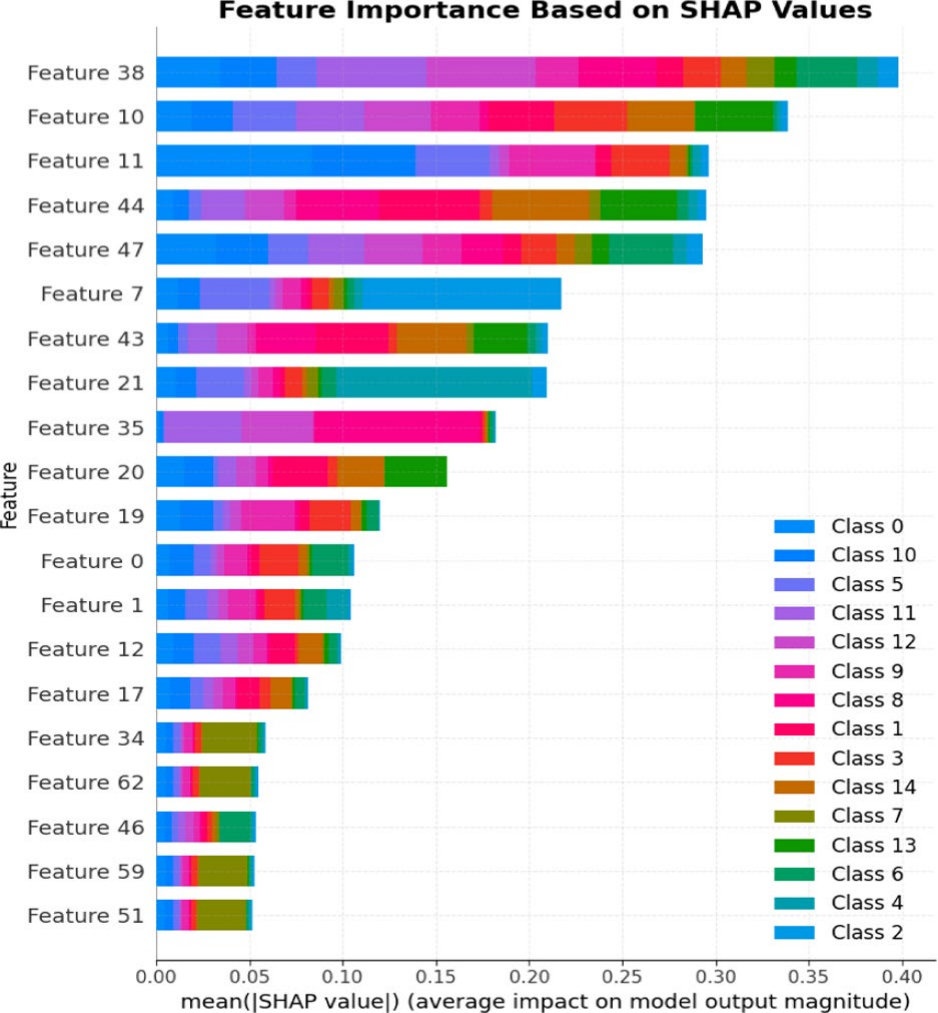
AdaBoost	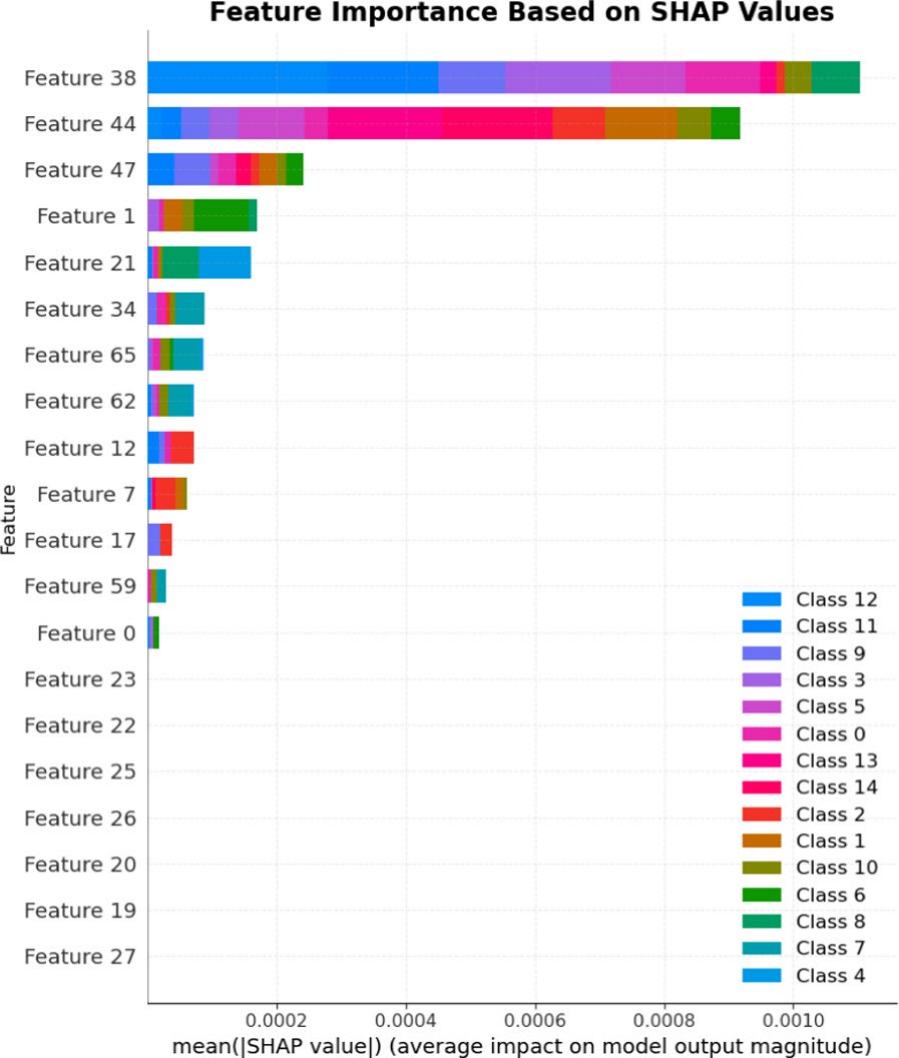	XGboost	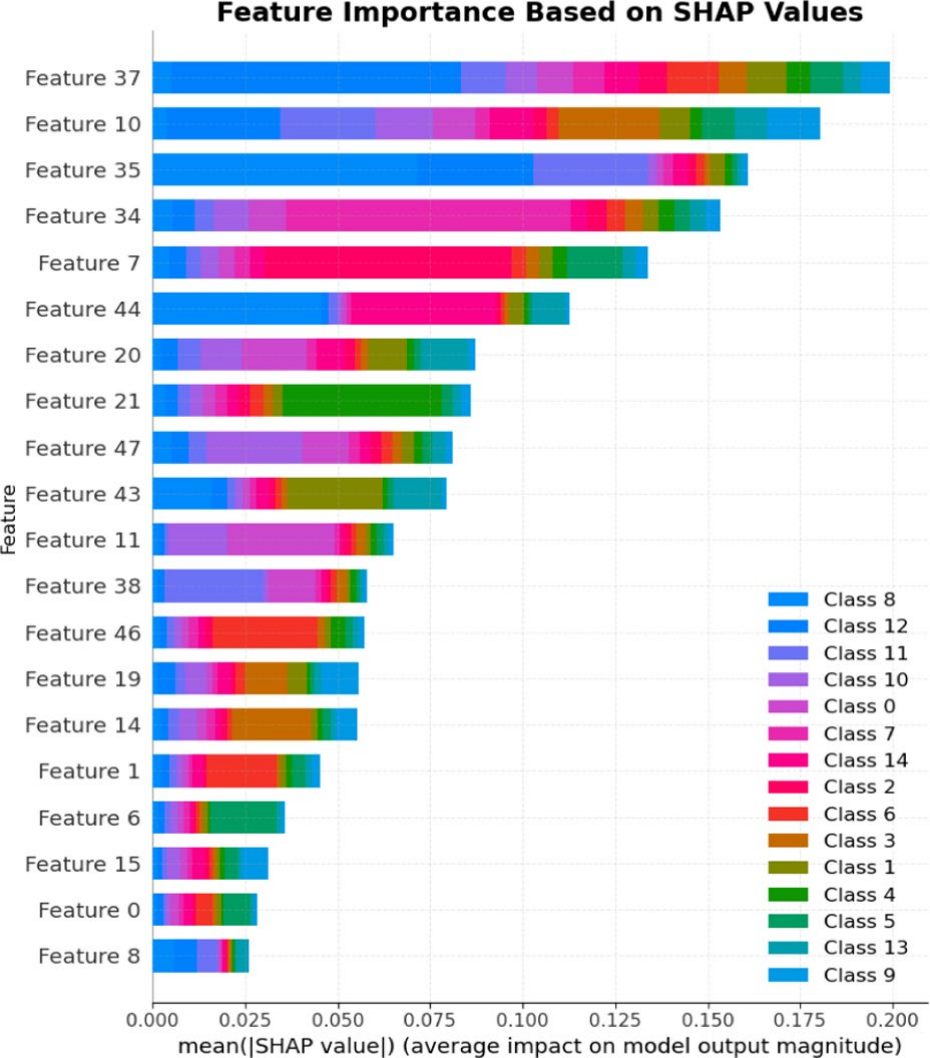
LGBM	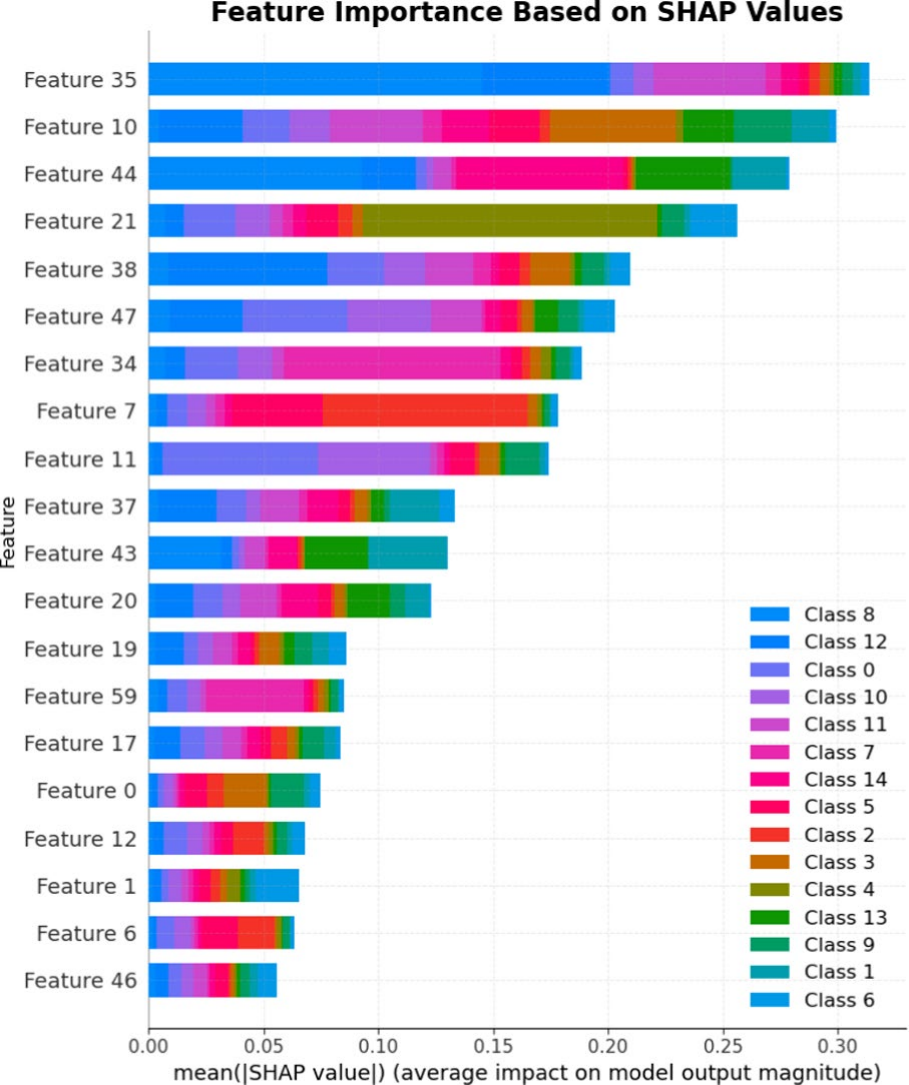	Extra tree	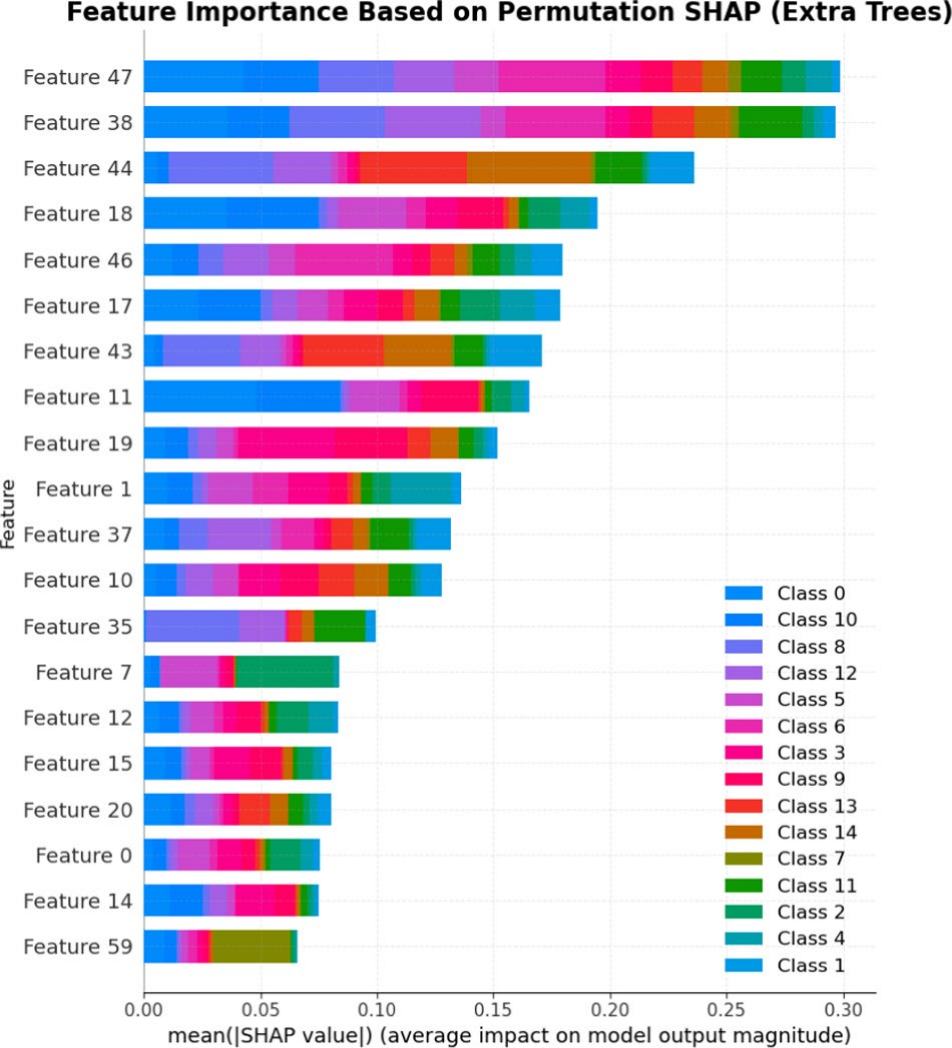
Stacked Ensemble_1	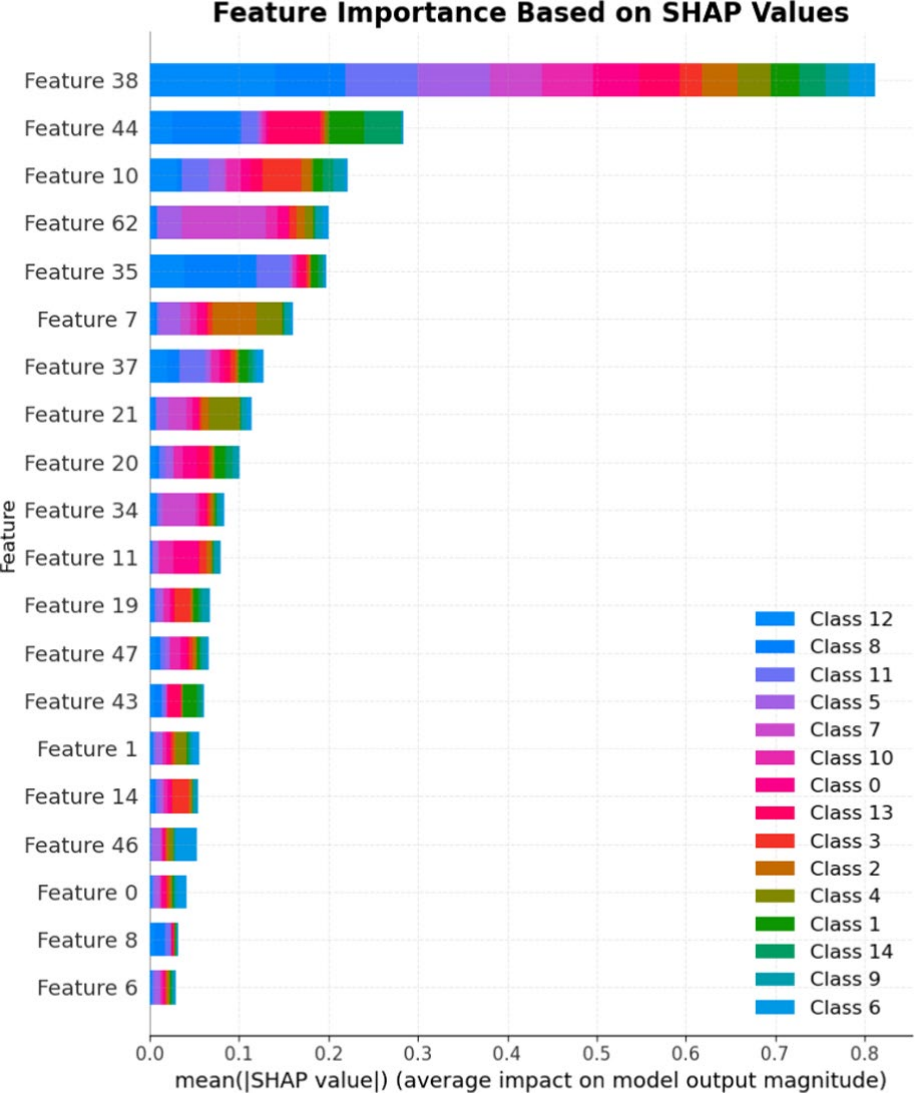	Stacked Ensemble_2	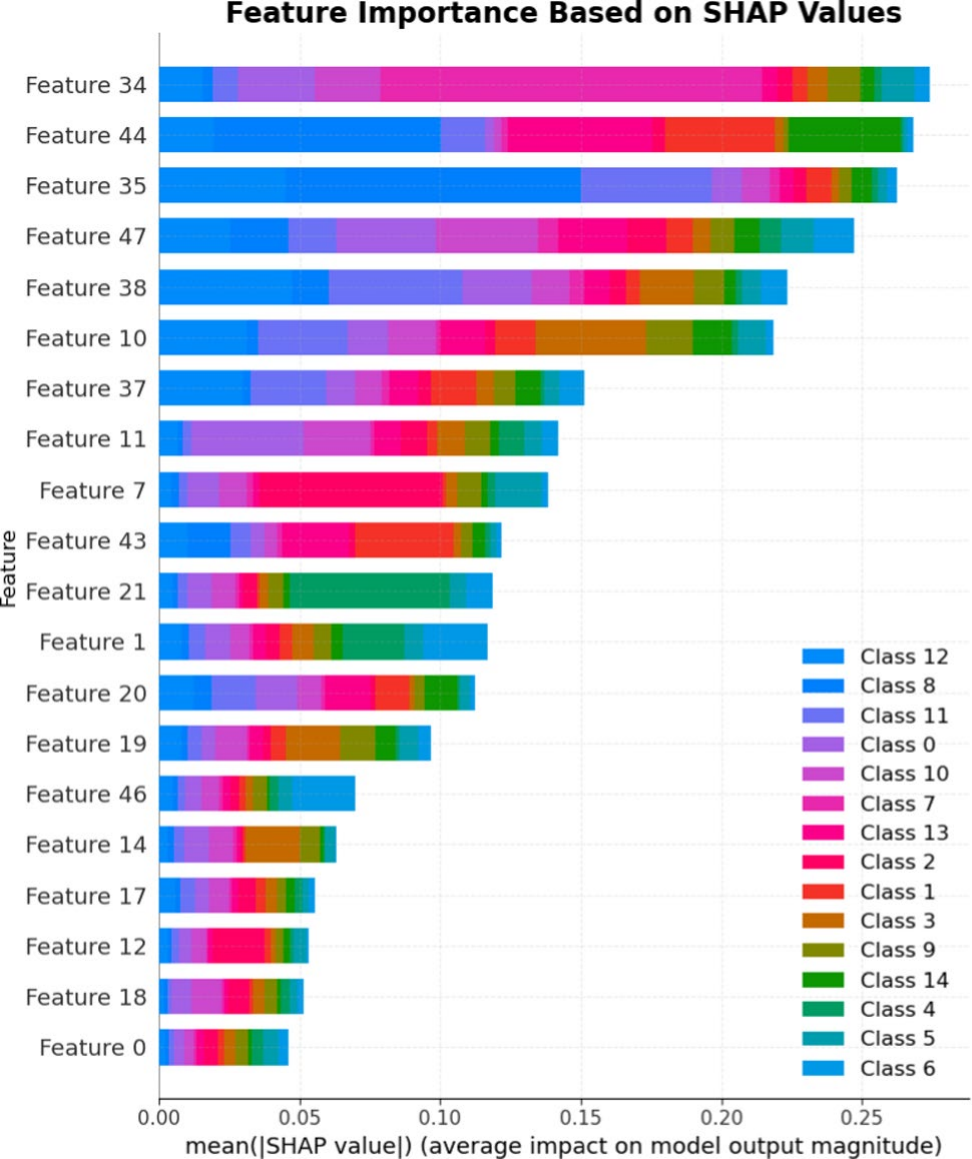

This comprehensive SHAP analysis offers a transparent and detailed understanding of how different models arrive at their predictions, highlighting both common underlying data patterns and algorithm-specific learning strategies. These insights are invaluable for model debugging, feature engineering, and enhancing overall trust and interpretability in complex machine learning systems.

## 8 Generalization of the model performance

CICIDS2017 dataset contains benign samples and the most up-to-date common attacks, resembling true real-world data (Canadian Institute of Cybersecurity, 2017). Network attacks feature 62 columns and 183,910 instances. The dataset includes network traffic in both packet-based and bidirectional flow-based formats. For this dataset, the abstract behavior of 25 users was modeled based on HTTP, HTTPS, FTP, SSH, and email protocols. To validate Stacked Ensemble_1 and 2 for model generalization, we chose a dataset similar to the one used in our work. The two stacked ensemble models were generalized on a new, unseen dataset for attack classification, which included eight attacks: Brute Force FTP, Brute Force SSH, DoS, Heartbleed, Web Attack, Infiltration, Botnet, and DDoS.

### 8.1 Data preprocessing

In this stage, several data cleaning and transformation steps were applied to the CICIDS2017 dataset to ensure data quality and consistency. First, duplicate records were identified and removed to eliminate redundant entries that could bias the model. Then, the missing values in the numerical features ‘Flow_Bytes/s’ and ‘Flow_Packets/s’ were filled using their respective median values, as the median is less affected by outliers and provides a robust estimate of central tendency. Infinite values, both positive and negative, were then replaced with NaN (Not a Number) to handle computational anomalies, as these “infinite” values can cause problems in analysis and model training, where most algorithms cannot handle them (Table 30).

**Table 30:** Data preprocessing for CICIDS2017 generalization task.

Before data preprocessing	After data preprocessing
2,695,162	2,661,918

After cleaning, the dataset’s categorical target variable (label) was analyzed to identify different types of network traffic, including both benign and various attack categories. To simplify the analysis, attack labels were grouped into broader categories (e.g., DoS, DDoS, Brute Force, Web Attack, etc.) using a mapping dictionary, and a new column named Attack Type was created to store these grouped labels. Finally, the Attack Type column was numerically encoded using a label encoder, producing a new feature (Attack Number) suitable for algorithms (Table 31).

**Table 31:** Feature mapping for target variable.

Actual name of the attack	Number of records	Mapping	Number of records	Label encoder
Benign	2,063,255	Benign	2,063,255	0
DdoS	128,016	DDoS	128,016	3
DoS Hulk	172,849	DoS	193,733	4
DoS GoldenEye	10,280			
DoS slowloris	5,376			
DoS Slowhttptest	5,228			
PortScan	90,819	Port Scan	90,819	7
FTP-Patator	5,933	Brute Force	9,152	2
SSH-Patator	3,219			
Bot	1953	Bot	1953	1
Web attack brute force	1,470	Web Attack	2,143	8
Web attack XSS	652			
Web attack Sql Injection	21			
Infiltration	36	Infiltration	36	6
Heartbleed	11	Heartbleed	11	5

### 8.2 Feature mapping

During the feature engineering stage, a correlation analysis was performed to identify features that exhibited a positive relationship with the target variable, Attack Number. A correlation matrix was computed, and attributes with correlation coefficients greater than zero and less than one were selected as positively correlated features, indicating their potential relevance in predicting attack behavior. Additionally, columns containing only a single unique value were identified and removed from the dataset, as such features do not contribute any variability or discriminative power to the model. Eliminating these constant-value columns helps reduce data redundancy and improves computational efficiency during training (Table 32).

**Table 32:** Correlation matrix.

All the features
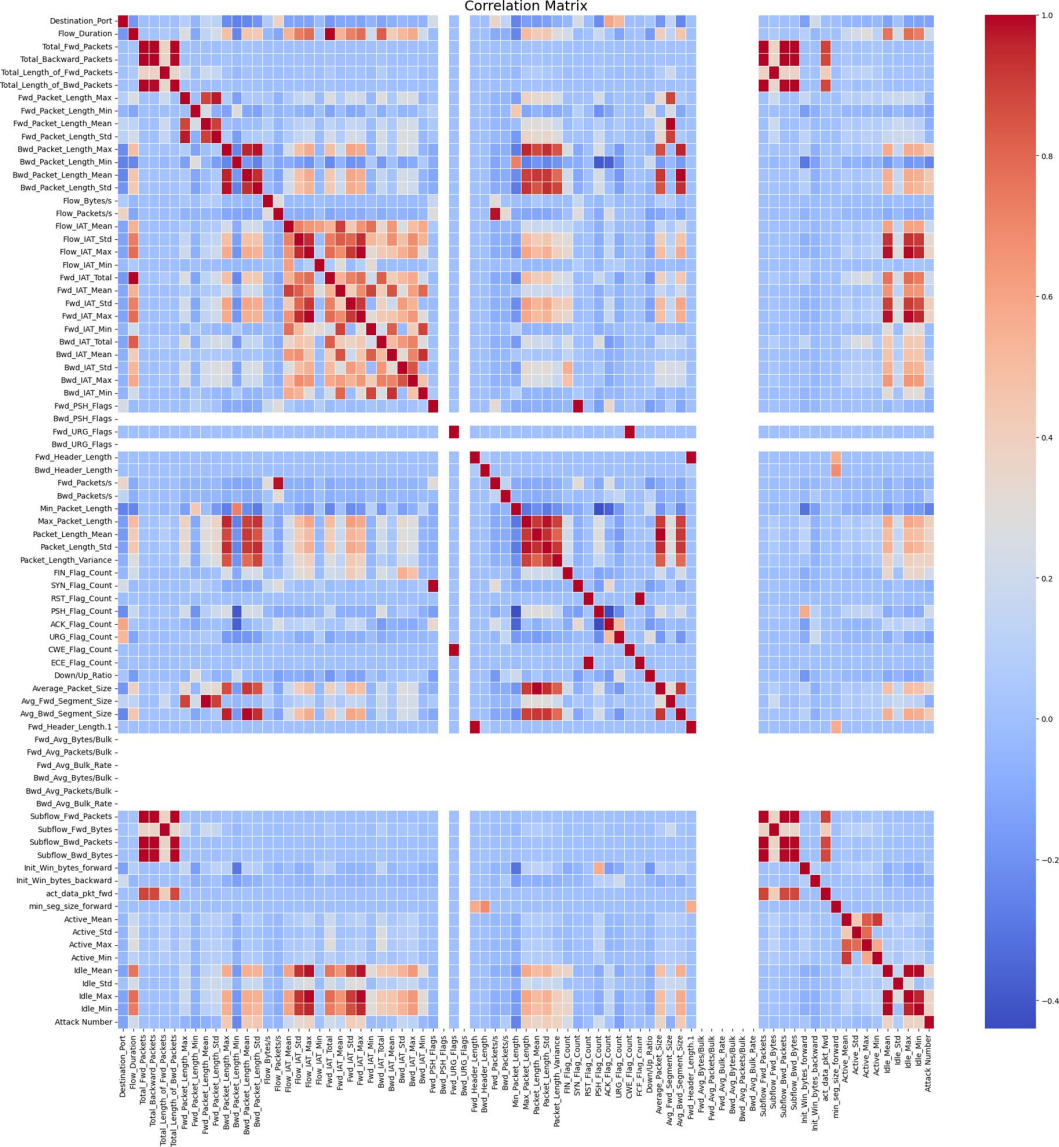

Dropped features	
1	Bwd_PSH_Flags
2	Bwd_URG_Flags
3	Fwd_Avg_Bytes/Bulk
4	Fwd_Avg_Packets/Bulk
5	Fwd_Avg_Bulk_Rate
6	Bwd_Avg_Bytes/Bulk
7	Bwd_Avg_Packets/Bulk
8	Bwd_Avg_Bulk_Rate

Features with positive correlation with attack number		
No	Features	Correlation value
1	1. Flow_Duration	0.21
2	Bwd_Packet_Length_Max	0.43
3	Bwd_Packet_Length_Mean	0.43
4	Bwd_Packet_Length_Std	0.45
5	Flow_IAT_Mean	0.18
6	Flow_IAT_Std	0.33
7	Flow_IAT_Max	0.38
8	Flow_IAT_Min	0.01
9	Fwd_IAT_Total	0.21
10	Fwd_IAT_Mean	0.15
11	Fwd_IAT_Std	0.41
12	Fwd_IAT_Max	0.38
13	Bwd_IAT_Mean	0.01
14	Bwd_IAT_Std	0.16
15	Bwd_IAT_Max	0.12
16	Bwd_Packets/s	0.07
17	Max_Packet_Length	0.4
18	Packet_Length_Mean	0.36
19	Packet_Length_Std	0.41
20	Packet_Length_Variance	0.38
21	FIN_Flag_Count	0.23
22	PSH_Flag_Count	0.21
23	ACK_Flag_Count	0.03
24	Average_Packet_Size	0.36
25	Avg_Bwd_Segment_Size	0.43
26	Init_Win_bytes_forward	0.03
27	Active_Mean	0.01
28	Active_Min	0.02
29	Idle_Mean	0.38
30	Idle_Std	0.08
31	Idle_Max	0.38
32	Idle_Min	0.37

To further reduce dimensionality and improve computational efficiency, Incremental Principal Component Analysis (IncrementalPCA) was applied to the scaled feature set. IncrementalPCA was chosen over standard PCA due to its ability to process large datasets in smaller batches, thereby reducing memory usage while preserving essential variance information. The number of principal components was set to half the number of original features to maintain a balance between information retention and dimensionality reduction. The model was trained iteratively using mini-batches of 500 samples, and the transformed feature space was generated to represent the principal components (PCs). A new dataset was created from these components, with each principal component labeled sequentially (e.g., PC1 and PC2), and the corresponding *Attack Type* values were appended to preserve the target variable for subsequent modeling.

### 8.3 Result and analysis

The (Stacked Ensemble_1 and Stacked Ensemble_2) models were successfully generalized on the new dataset. The results indicate their robustness in accurately classifying diverse attack patterns not seen during training. The high-performance metrics on CICIDS2017 suggest that these stacked ensemble models are capable of effectively detecting multiple attack types in real-world network environments, confirming their potential utility for ongoing cybersecurity defense, as shown in Table 33. Stacked Ensemble_2 demonstrated better model generalization over Stacked Ensemble_1, performing exceptionally well across all metrics and making it suitable for cyberattack detection.

**Table 33:** Model generalization.

Models	Accuracy	Precision	Recall	F1 Score	TPR	FPR	AUROC
Stacked_Ensemble_1	99.96%	99.96%	99.96%	99.96%	85%	0.01%	99.00%
Stacked Ensemble_2	100%	100%	100%	100%	98%	0%	100%

Classes	Classification report–Stacked Ensemble_1				Classification report–Stacked Ensemble_2			
	Precision	Recall	F1-score	Support	Precision	Recall	F1-score	Support
BENIGN	100%	100%	100%	412,651	100%	100%	100%	412,651
Bot	99%	90%	94%	391	100%	99%	100%	391
Brute force	100%	99%	100%	1830	100%	100%	100%	1830
DDoS	100%	100%	100%	25,603	100%	100%	100%	25,603
Dos	100%	100%	100%	38,747	100%	100%	100%	38,747
Heartbleed	100%	50%	67%	2	100%	100%	100%	2
Infiltration	67%	29%	40%	7	75%	86%	80%	7
Port scan	100%	100%	100%	18,164	100%	100%	100%	18,164
Web attack	100%	100%	100%	429	100%	100%	100%	429
Model performance result after training								
Accuracy			100%	497,834			100%	497,834
Macro avg	96%	85%	89%	497,834	97%	98%	98%	497,834
Weighted Avg	100%	100%	100%	497,834	100%	100%	100%	497,834

Additionally, we conducted an experiment to evaluate the impact of synthetic oversampling on model behavior by comparing two pipelines:

- Training on the original imbalanced dataset (No SMOTE)
- Training after applying SMOTE to the training set only (SMOTE).

Because the full dataset is extremely large and contains classes with very low sample counts, we first constructed a balanced and computationally manageable subset by capping large classes at 100,000 samples while retaining all minority classes with fewer than 200 samples. This approach ensured that each class had enough instances for both stratified splitting and SMOTE to operate without errors. We included k_neighbors = 3 to avoid synthetic sample generation errors in minority classes. The comparison included accuracy, precision, recall, F1-score, AUROC, and particularly the false-positive rate for the benign class (Table 34).

**Table 34:** Evaluation of the synthetic impact.

Stacked Ensemble_1: without SMOTE					
Accuracy	Precision	Recall	F1 Score	AUROC	Benign FPR
Data subset size: (404,114, 35)					
99.87%	99.87%	99.87%	99.87%	99.93%	0.240%
Data Size: (720,000, 35)					
99.82%	99.81%	99.82%	99.81%	95.55%	0.195%

Stacked Ensemble_2: without SMOTE					
Accuracy	Precision	Recall	F1 Score	AUROC	Benign FPR
Data Subset Size: (404,114, 35)					
99.99%	99.99%	99.99%	99.99%	100%	0.010%
Data Size: (720,000, 35)					
99.98%	99.98%	99.98%	99.97%	97.62%	0.035%

Many tree-based ensemble methods inherently handle class imbalance through mechanisms such as weighted impurity reduction, adaptive boosting of minority errors, and probabilistic leaf estimation, which enable them to learn minority-class patterns without requiring explicit oversampling. Because these models naturally focus on the minority classes, adding synthetic samples through SMOTE does not introduce additional useful information; instead, it may introduce noise or overly smoothed synthetic points. This disrupts decision boundaries and can increase false positives, especially for the benign class. Consequently, for strong ensemble learners that already manage imbalance effectively, SMOTE provides no practical gain and often reduces overall performance, as demonstrated in our experiments.

## 9 Benchmarking of results

Table 35 shows benchmarking of the proposed work against other related work in the classification of attacks, which is outlined below.

**Table 35:** Benchmarking of results.

Work	Objective	Dataset	Algorithm
Abedin et al. (2020)	Phishing attack detection	32 attributes and 11,504 instances. The dataset contains both phishing and legitimate website data	Three supervised ML algorithms: KNN, LR, and RFC for binary classification (phishing and legitimate). RFC: P 97%, R 99%, F1 97% LR: P 83%, R 96%, F1 89% KNN: P 91%, R 94%, F1 95%
Pithawala et al. (2021)	Short uniform resource locators	Dataset 1780 entries with 19 features related to phishing and non-phishing URLs.	Three ML algorithms: naive Bayes (NB), Logistic Regression (LR), and Random Forest Classifier (RFC) NB accuracy = 99.4% RF accuracy = 98% LR accuracy = 96%.
Ahmed and Tjortjis (2022)	IoT-botnet attack detection using real-time heterogenous data	Dataset: 461,043 samples, with 65.07% normal traffic and 34.93% malicious traffic. The dataset consists of 43 features across 6 categories: connection activity, DNS, SSL, statistical, HTTP, and violation activity	DT, RF, KNN, XGB outperformed LR and GNB, with an accuracy over 99% and F1-scores of 0.98–0.99 for binary classification – malicious and normal
Mubarak et al. (2021)	ICS cyber attack detection using cyber-kit datasets	Dataset: Network traffic data from different ICS protocols, such as Modbus/TCP, Ethernet/IP, and IEC 61850, along with a normal baseline and diverse industrial hacking scenarios. Deep packet inspection (DPI) was used to extract metadata features from network traffic data, and the final dataset matrix is (30,608,16) with two classes- Secure and insecure	Ensemble ML including both traditional (LR, KNN, NB, RF, ANN, SVM, DT) and DL (RNN, LSTM) for binary classification only (secure and insecure traffic). The ensemble approach resulted in 99.91% prediction accuracy.
Yeboah-Ofori (2020)	Classification of malware attacks	Microsoft Malware Prediction dataset: 4000 entries with 64 columns representing various metadata about the machines and malware infection as a binary class.	The C4.5 and C5.0 variants of the DT algorithm Accuracy = 83%
Syafiuddin et al. (2023)	Detection Syn flood and UDP Lag attacks based on AdaBoost	CICDDoS2019 dataset, which is a dataset of network traffic containing simulated DDoS attacks on 25 different network users	AdaBoost algorithm outperformed other machine learning algorithms (RFC, Simple Logistic, and REP Tree) with values above 47.2% for detecting SYN flood and UDP lag attacks which is binary classification
Saharkhizan et al. (2020)	Ensemble of deep RNN for IoT cyber attacks	Dataset: Modbus network traffic, including 5 types of traffic (clean, man-in-the-middle attack, Ping DDoS Flood attack, Modbus Query Flood attack, and TCP SYN DDoS Flood attack). The dataset was captured in pcap files and pre-processed to extract 83 features totaling 5,859,085 sample.	Integration of LSTM models into an ensemble model. Then aggregate output using a DT for detection of cyberattack as binary classifier. Ensemble of LSTM accuracy = 99% for a window size of 40 packets for Modbus traffic toward cyberattack detection as a binary classifier.
Alasmari et al. (2023)	CNN-LSTM based Approach for DDoS Detection	CI-CDDoS2019 dataset contains network traffic data with 400,000 datapoints and 12 different types of DDoS attacks labeled as benign and DDoS.	1D CNN-LSTM model has been used and achieved an accuracy of 99.51% in detecting DDoS attacks, outperforming the other ML algorithms tested, which are naive Bayes (96%), SVM (97.40%), Bayes Net (97%), Logistic Regression (97%), and Random Forest (99.01%).
Canadian Institute of Cybersecurity (2017)	Port-scanning attack detection	CICIDS2017 dataset: Network attacks with 62 columns and 183,910 instances. Includes network traffic in packet-based and bidirectional flow-based format.	Five ML algorithms: DT, RF, AdaBoost, KNN, and SVM for port scanning attack as binary classification. DT accuracy = 99.84%, RF accuracy = 99.75% AdaBoost accuracy = 99.64%, KNN accuracy = 99.84% SVM accuracy = 89.61
Huynh et al. (2023)	DOS Attack Detection	Two datasets - a real-time dataset captured using a packet sniffer on an ESP32 microcontroller, and the CICIoT2023 dataset which contains a wider variety of DoS and DDoS attacks for classification as Benign and DDoS	Two algorithms: SVM and LR - for binary classification. Both SVM and LR accuracy = 99%
Ferrag et al. (2022)	Baseline model (Edge-IIoT dataset)	The dataset consists of 157,800 samples across 15 types of attacks which are categorized into five threat types, including DoS/DDoS attacks, information Gathering, Man-in-the-Middle attacks, Injection attacks, and Malware attacks.	Three algorithms: Decision Tree, KNN, Random Forest, and DNN employed for classification of 15 attacks. Results in maximum accuracy of 94.61% with Deep Neural, followed by 79.18% for KNN, 77.61% for SVM, 80.83% for RFC, and 67.11% for DT.
Proposed work (Edge IIoT dataset)	Proposed work	The dataset consists of 157,800 samples across 15 types of attacks which are categorized into five threat types, including DoS/DDoS attacks, Information Gathering, Man-in-the-Middle attacks, Injection attacks, and Malware attacks.	Stacked Ensemble_2: Accuracy = 97% Train time = 54.24 min Test Time = 0.97 min Stacked Ensemble _1: Accuracy = 96% Train Time = 0.99 min Test time = 6.47 min Model Generalization on CICDDOS 2017 Stacked Ensemble 1: Accuracy = 99% Precision = 99% Recall = 99% F1 score = 99% TPR = 85% FPR = 0.001%, AUROC = 99% Stacked Ensemble_2: Accuracy = 100% Precision = 100% Recall = 100% F1 Score = 100% TPR = 98% FPR = 0.00 AUROC = 100%

From benchmarking analysis, most of the work has focused either on binary classification for one type of attack, such as Phishing, Malware, Ransomware, DoS, or Port scanning. In addition, one study concentrated on Modbus Network traffic, specifically for various attacks like man-in-the-middle, Ping DDoS Flood, Modbus Query Flood, and TCP SYN DDoS Flood, targeting cyberattack detection. The works reported have utilized limited datasets and larger datasets, achieving an accuracy of 98–99% with individual classifiers such as LSTM, 1D CNN-LSTM, and an Ensemble of LSTM with Decision Tree Classifier. Some studies have employed ensemble models like Random Forest and Adaboost algorithms. A baseline model based on the Edge-IIOT dataset provided 94.61% accuracy with a Deep Neural model, compared to other models like Decision Tree, KNN, SVM, and Random Forest. None of the studies have utilized advanced ensemble models like stacked ensemble models with linear and non-linear classifiers, combined with model optimization, to achieve higher accuracy and reduced computational operations for multi-class classification rather than binary.

This has been addressed in our proposed model, which employs a stacked ensemble that includes linear and non-linear decision boundaries (Logistic Regression, XGBoost, LGBM, Extra Trees as base models, and RFC as the final estimator) with good performance and reduced computation time. We have optimized models to reduce computation time, making our work stand out from other models. Furthermore, our use of a stacked ensemble is novel compared to existing ensembles like Bagging, Boosting, and Random Forest, demonstrating improved performance against the baseline model and reduced computation time. Lastly, our proposed stacked ensemble performed well in classifying multi-class attacks compared to other works that focused on binary classification or a single attack category, such as DDoS or Modbus network traffic. Our models were also generalized on the CICDOS 2017 dataset for classifying eight attacks, which is similar to our IIoT dataset, resulting in excellent performance by Stacked Ensemble_2, as tabulated in Table 24.

## 10 Conclusion and future work

In this study, the dataset contains a total of 157,800 data points representing 15 types of complex cyberattacks targeting the Oil and Gas ICS and IoT domain. We performed a comprehensive data preprocessing step to extract the most important features, as the data points were collected from network traffic containing diverse data types, requiring careful feature engineering to obtain the most useful information. We then analyzed and compared the performance of individual machine learning algorithms and ensemble learning classifiers, which included Decision Tree, Random Forest, Bagging, Boosting, Extra Tree, and two proposed stacked ensemble models. The results showed that the proposed stacked ensemble learning approach, referred to as stacked ensemble_2, performed better than the stacked ensemble, other ensemble models, and individual machine learning models, achieving an average accuracy of 97% in detecting and classifying the Oil and Gas ICS and IoT attacks. The precision, recall, and F1 score also performed quite well among stacked ensembles compared to other ensemble models and individual classifiers. Furthermore, the stacked ensemble models that excelled on the IIOT dataset were generalized on the CICDOS 2017 dataset, demonstrating excellent performance with stacked ensemble_2 compared to stacked ensemble_1, achieving 100% accuracy and 100% AUROC.

To further enhance the model’s performance, we utilized the Grid Search method to optimize the hyperparameters of the ensemble learning models and ML models. This optimization step improved the models’ ability to accurately detect and classify the various types of cyberattacks targeting the Oil and Gas ICS and IoT systems. Overall, this study demonstrates the effectiveness of the ensemble learning approach in enhancing the detection and classification of complex cyber threats in the Oil and Gas ICS and IoT domain, with the optimized models achieving highly promising results.

Future work for this study will focus on enhancing security measures for ICS and IoT devices by improving anomaly detection and vulnerability assessment, with special attention given to refining intrusion detection algorithms in the oil and gas sector. We aim to expand feature engineering to include more contextual data from IoT devices to provide deeper insights and improve model accuracy. Additionally, we will leverage deep learning techniques, such as LSTM, to enable the identification of complex patterns within large datasets.

Generative AI can also simulate attack scenarios for testing and enriching training data to enhance model robustness, thereby increasing the diversity of base classifiers and further improving detection effectiveness. The main motivation behind this study is to assist oil and gas engineers and experts in identifying attacks on ICS and IoT computing systems, encouraging them to take appropriate countermeasures early on with the help of high-accuracy models. These models can help detect malicious attacks that may evade software cybersecurity tools, posing a significant security risk to all interconnected systems and devices in the oil and gas sector. As reliance on these technologies grows, advanced methods for safeguarding ICS and IoT environments become essential, ensuring the safety and reliability of operations in our increasingly digital world.

## Data Availability

The original contributions presented in the study are included in the article/supplementary material, further inquiries can be directed to the corresponding author/s.
