# Correction: Optimized ensemble machine learning model for cyberattack classification in industrial IoT

**DOI:** 10.3389/frai.2026.1786635

**Published:** 2026-02-10

**Authors:** Batool Alabdullah, Suresh Sankaranarayanan

**Affiliations:** College of Computer Sciences and Information Technology, Department of Computer Science, King Faisal University, Al Ahsa, Saudi Arabia

**Keywords:** cyberattack, ensemble learning, industrial control systems, industrial internet of things, internet of things, machine learning, malicious behavior, oil and gas

In the published article, there was an error regarding the affiliation for Batool Alabdullah and Suresh Sankaranarayanan. The affiliation should be updated to *College of Computer Sciences and Information Technology, Department of Computer Science, King Faisal University, Al Ahsa, Saudi Arabia*.

The original article has been updated.

